# Human Trisomic iPSCs from Down Syndrome Fibroblasts Manifest Mitochondrial Alterations Early during Neuronal Differentiation

**DOI:** 10.3390/biology10070609

**Published:** 2021-06-30

**Authors:** Nunzia Mollo, Matteo Esposito, Miriam Aurilia, Roberta Scognamiglio, Rossella Accarino, Ferdinando Bonfiglio, Rita Cicatiello, Maria Charalambous, Claudio Procaccini, Teresa Micillo, Rita Genesio, Gaetano Calì, Agnese Secondo, Simona Paladino, Giuseppe Matarese, Gabriella De Vita, Anna Conti, Lucio Nitsch, Antonella Izzo

**Affiliations:** 1 Department of Molecular Medicine and Medical Biotechnology, University of Naples Federico II, 80131 Naples, Italy; nunzia.mollo@unina.it (N.M.); matteo.esposito@unina.it (M.E.); mi.aurilia@studenti.unina.it (M.A.); roberta.scognamiglio8@studenti.unina.it (R.S.); rossella.accarino@unina.it (R.A.); rita.cicatiello@unina.it (R.C.); rgenesio@unina.it (R.G.); spaladin@unina.it (S.P.); giuseppe.matarese@unina.it (G.M.); gdevita@unina.it (G.D.V.); anconti@unina.it (A.C.); nitsch@unina.it (L.N.); 2 CEINGE-Biotecnologie Avanzate s.c.ar.l., 80145 Naples, Italy; ferdinando.bonfiglio@unina.it; 3 Department of Chemical, Materials and Production Engineering, University of Naples Federico II, 80125 Naples, Italy; 4 Institute of Experimental Endocrinology and Oncology “G. Salvatore”, National Research Council, 80131 Naples, Italy; m.charalambous@ieos.cnr.it (M.C.); claudio.procaccini@cnr.it (C.P.); g.cali@ieos.cnr.it (G.C.); 5 Neuroimmunology Unit, IRCCS, Fondazione Santa Lucia, 00143 Rome, Italy; teresa.micillo2@unina.it; 6 Department of Neuroscience, Reproductive and Odontostomatological Sciences, University of Naples Federico II, 80131 Naples, Italy; secondo@unina.it

**Keywords:** Down syndrome, induced pluripotent stem cells, neural differentiation, mitochondrial dysfunction

## Abstract

**Simple Summary:**

Down Syndrome, which is due to the presence of three copies of chromosome 21, always presents with mental retardation, possibly caused by defects in the development of neurons. In recent years, it has been shown that cells and tissues in Down syndrome manifest alterations in the function of mitochondria, the organelles that provide energy to cells. We hypothesized that mitochondrial dysfunction might contribute to the defect in neuronal cell development. To test this hypothesis, we generated a model of stem cells that, upon specific treatments, are capable of giving rise to neuronal cells, as evidenced by the synthesis of specific proteins. We observed that stem cells derived from Down syndrome individuals, after 21 days of growth in an artificial system, had an abnormal tendency to develop as glial cells, compared with control cells. As early as day 7 of culture, the trisomic cells also exhibited defects in mitochondrial function, such as anomalies in their calcium level, oxygen free radicals, oxygen consumption, and synthesis of ATP, a molecule that is critical in energy conversions. These results indicate that alterations in neuronal development and mitochondrial function occur early in this model, which we think is suitable for answering further questions.

**Abstract:**

Background: The presence of mitochondrial alterations in Down syndrome suggests that it might affect neuronal differentiation. We established a model of trisomic iPSCs, differentiating into neural precursor cells (NPCs) to monitor the occurrence of differentiation defects and mitochondrial dysfunction. Methods: Isogenic trisomic and euploid iPSCs were differentiated into NPCs in monolayer cultures using the dual-SMAD inhibition protocol. Expression of pluripotency and neural differentiation genes was assessed by qRT-PCR and immunofluorescence. Meta-analysis of expression data was performed on iPSCs. Mitochondrial Ca^2+^, reactive oxygen species (ROS) and ATP production were investigated using fluorescent probes. Oxygen consumption rate (OCR) was determined by Seahorse Analyzer. Results: NPCs at day 7 of induction uniformly expressed the differentiation markers PAX6, SOX2 and NESTIN but not the stemness marker OCT4. At day 21, trisomic NPCs expressed higher levels of typical glial differentiation genes. Expression profiles indicated that mitochondrial genes were dysregulated in trisomic iPSCs. Trisomic NPCs showed altered mitochondrial Ca^2+^, reduced OCR and ATP synthesis, and elevated ROS production. Conclusions: Human trisomic iPSCs can be rapidly and efficiently differentiated into NPC monolayers. The trisomic NPCs obtained exhibit greater glial-like differentiation potential than their euploid counterparts and manifest mitochondrial dysfunction as early as day 7 of neuronal differentiation.

## 1. Introduction

Down syndrome (DS) is caused by trisomy of chromosome 21 (Hsa21). The DS phenotype, which possibly results from the interaction between the over-expression of genes mapping to the trisomic chromosome and the subsequent dysregulation of genes mapping to different chromosomes, is always characterized by neuro-developmental anomalies and early neuro-degenerative processes [1].

Impaired mitochondrial function in DS is widely documented. Reduced mitochondrial redox activity and membrane potential have been observed in DS astrocytes and neuronal cultures [2], and decreased protein levels of mitochondrial electron transport enzymes have been observed in cerebellar and brain regions of DS subjects [3]. In addition, a decrease in mitochondrial membrane potential and ATP production, and an increase in reactive oxygen species (ROS) were demonstrated in the brain of a DS model, the Ts1Cje mouse [4]. The mitochondrial morphology is consistently altered in DS neurons, which exhibit increased fragmentation [5]. Adult hippocampal progenitors from the Ts65Dn mouse model of DS display altered mitochondrial bioenergetics and reduced cell energy status. In the same cells, the mitochondrial biogenesis program is impaired. This has been assessed by low levels of the mitochondrial DNA and of the co-activator PGC-1α and transcription factors NRF-1 and T-FAM, which regulate nuclear- and mitochondrial-encoded gene transcription, and mitochondrial DNA replication [6]. Many studies in DS hippocampus and other tissues have reported aberrant hyperactivation of the AKT/mTOR signaling pathway suggesting that imbalances in autophagy flux regulation in DS leads to negative effects on mitochondrial turnover [7]. All these findings indicate that trisomy of Hsa21 (T21) affects mitochondrial function and, as a possible consequence, the production of ROS in neural tissues.

The investigation of the molecular mechanisms by which T21 causes the DS phenotype is hampered by the lack of suitable models. Transgenic mice have proved to be imperfect models, and human tissues at the appropriate developmental stages are difficult to obtain. Therefore, new models would be useful to bypass these drawbacks and stem cells could represent an adequate tool to reproduce stages otherwise difficult to obtain. Indeed, stem cells derived from individuals affected by DS can be induced to differentiate into cell types targeted by the disease [8]. This makes it possible to investigate very early alterations that occur during cell differentiation, mimicking the specific developmental in vivo changes that contribute to the phenotype. A cell culture model would also be useful to validate the role of candidate genes and networks possibly involved in determining the DS neuronal phenotype, and to determine whether they can be effectively targeted by therapeutic approaches.

Based on these assumptions, we planned to use human induced pluripotent stem cells derived from DS subjects to investigate the molecular and biological consequences of T21 on mitochondrial phenotype and neurogenesis.

The main focus of this study was to establish a novel and robust human model to investigate (i) if Hsa21 affects neuronal differentiation; (ii) if and when the mitochondrial dysfunction occurs during the differentiation process. To achieve these goals, we differentiated trisomic (T21-iPSC) and euploid isogenic iPSCs (Eu-iPSCs) into neural precursor cells (NPCs) using the dual-SMAD inhibition protocol [9]. The ability of these cells to differentiate into neural precursors and subsequently into neurons has been previously demonstrated [10,11,12]. The advantage of using these iPSCs is that they are isogenic, being derived from retroviral reprogramming of a culture of human fibroblasts mosaic for Hsa21 trisomy. Neuronal cell markers and mitochondrial phenotype were investigated before, during, and after induction of neurogenesis in trisomic lines and euploid isogenic controls to evaluate the effects of Hsa21 trisomy upon neural differentiation.

**Figure 1:**
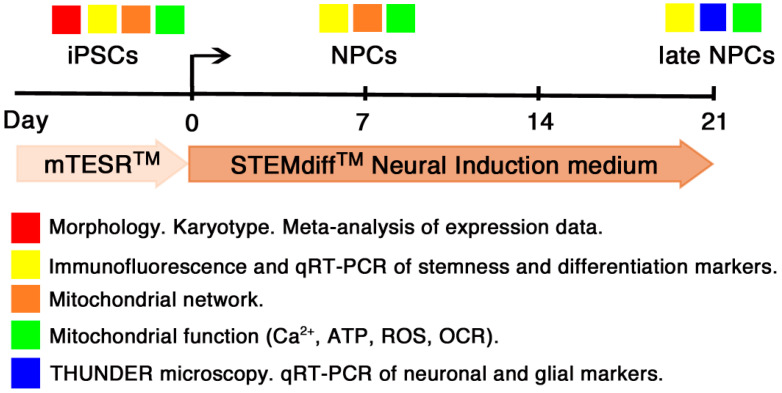
Schematic representation of the protocol used to characterize NPCs obtained from Eu- and T21-iPSCs. Euploid and trisomic iPSC lines were maintained in mTESR medium. For neural induction, cells were plated in STEMdiff Neural Induction medium and splitted at days 7, 14, and 21. Timeline is annotated with colored squares indicating the timepoint of each performed analysis.

We then proceeded to the evaluation of mitochondrial functionality in undifferentiated and differentiated T21-iPSCs. First of all we retrieved, from a public database, gene expression data of undifferentiated T21-iPSCs and euploid controls in order to investigate, through a meta-analysis of a larger set of data, whether and how the expression profile of mitochondria-related genes is perturbed in T21-iPSCs. Lastly, based on the results of the gene expression analysis, we investigated mitochondrial organization and bioenergetic features in euploid and trisomic iPSCs and in derived NPCs. Possible implications of a mitochondrial dysfunction on neural stem cell fate are discussed. A schematic presentation of the protocol used to generate and characterize NPCs from Eu-iPSCs and T21-iPSCs is illustrated in Figure 1.

## 2. Materials and Methods

### 2.1. iPSC Culture Conditions

Isogenic induced pluripotent stem cells, euploid UWWC1-DS2U (Eu-iPSCs) and Hsa21 trisomic UWWC1-DS1 and UWWC1-DS4 (T21-iPSCs), obtained from the skin biopsy of a 1-year-old subject with DS [9] (WiCell Research Institute, Madison, WI, USA), were cultured on a thin-layered basement membrane Geltrex™ (Gibco^TM^, Thermo Fisher Scientific, Waltham, MA, USA) and maintained with daily media changes using mTeSR1 (STEMCELL^TM^ Technologies, Cambridge, UK). Cells were passaged upon reaching confluency using a 50 mM EDTA solution, and care was taken to prevent the formation of differentiation foci in the culture.

### 2.2. Karyotype

Karyotyping was performed on iPSCs at passages 7–13 following standard cytogenetic procedures and according to the International System of Human Cytogenetic Nomenclature (ISCN) 2016, as previously described [13].

### 2.3. RNA Extraction and Quantitative RT-PCR 

Total RNA from each sample was extracted using TRIzol reagent (Gibco, Thermo Fisher Scientific) and was reverse-transcribed using the iScript cDNA Synthesis kit (Bio-Rad Laboratories, Inc., Hercules, CA, USA). Quantitative real time polymerase chain reaction (qRT-PCR) was performed using SsoAdvanced universal SYBR Green supermix on a Bio-Rad iCycler CFX96 Touch Real-Time PCR Detection System according to the manufacturer’s protocols. Primer pairs (MWG Biotech, Ebersberg, Germany) were designed using the Primer 3 software (https://bioinfo.ut.ee/primer3/, accessed on 4 November 2019) to obtain amplicons ranging from 100 to 150 base pairs. Primer efficiency was tested generating standard curves for each gene. QRT-PCR results are presented as relative mRNA levels normalized against *ACTIN* housekeeping gene as reference gene.

### 2.4. Neural Induction

Differentiation towards neural progenitors was achieved using the STEMdiff^TM^ Neural System following the monolayer culture protocol. In brief, iPSC colonies were detached using the Gentle Cell Dissociation Reagent (STEMCELL^TM^ Technologies) and plated on Geltrex-coated wells at a density of 2 × 10^5^ cells/cm^2^, in Neural Induction Medium (STEMCELL^TM^ Technologies) supplemented with SMAD inhibitors (STEMCELL^TM^ Technologies) and Y-27632 at 10 μM (Merck, Darmstadt, Germany), with daily medium changes. Cells were harvested at day 7 to perform qRT-PCR and immunofluorescence analyses and passaged two more times at a seeding density of 1.26 × 10^5^ cells/cm^2^ using StemPro^TM^ Accutase^TM^ Cell Dissociation reagent (Gibco, Thermo Fisher Scientific). Cells were harvested at day 21 for subsequent analyses.

### 2.5. Immunofluorescence

iPSCs at different stages of neural differentiation grown on coverslips were fixed as previously described [14] and permeabilized for 5 min in 1% Triton X-100 in PBS. Blocking was performed with 1% bovine serum albumin (BSA) in PBS for 30 min at room temperature. Alternatively, for the NESTIN/SOX2 experiments, cells were permeabilized for 30 min in 0.2% Triton X-100 in PBS and blocked in 5% BSA in PBS for 1 hr. Cells were incubated with primary antibodies for 2 h at RT, washed in 0.5% BSA in PBS, and then incubated with secondary antibodies for 30 min. Coverslips were finally mounted in 50% glycerol in PBS containing the DNA intercalator Hoechst 33,258 (Merck). Primary antibodies were: PAX6 (BioLegend, San Diego, CA, USA), OCT-3/4 (Santa Cruz Biotechnology, Santa Cruz, CA, USA), NESTIN (Millipore, Burlington, MA, USA), SOX2 (R&D Systems, Minneapolis, MN, USA), TOM20 (Proteintech, Rosemont, IL, USA). The secondary antibodies were Alexa Fluor^®^ 488 goat anti-mouse IgG (H + L) (Thermo Fisher Scientific) (green) and DyLight^®^ 550 goat anti-rabbit IgG (H + L) (ImmunoReagents, Raleigh, NC, USA) (red). Experiments were performed at least in triplicate.

Images were collected using confocal laser scanning microscope LSM 700 (Carl Zeiss, Jena, Germany) or the Leica Thunder Imaging System (Leica Microsystems).

For confocal immunofluorescence, analyses were carried out on an inverted and motorized microscope (Axio Observer Z.1) equipped with a 63 × 1.4 Plan-Apochromat objective. The attached laser-scanning unit (LSM 700 4 pigtailed laser 405-488-555-639) enabled confocal imaging. For excitation, 405, 488, and 555 nm lasers were used. Fluorescence emission was revealed by a main dichroic beam splitter and a variable secondary dichroic beam splitter. Triple staining fluorescence images were acquired separately using the ZEN Black 2012 software in the red, green, and blue channels at a resolution of 1024 × 1024 pixels, with the confocal pinhole set to one Airy unit and then saved in the TIFF format. Leica Thunder Imaging System was equipped with the Leica DFC9000GTC camera. Fluorescence LED light source and appropriate excitation and emission filters for Alexa Fluor-488, -546 or DAPI were used. Images were acquired using a semi-apochromat PL fluotar 20× (NA 0.4) objective lens, taking Z-slices from the top to the bottom of the cell by using the same setting (LED source power, exposure time) and the Small Volume Computational Clearing (SVCC) mode for the different experimental conditions.

### 2.6. Mitotracker Fluorescence 

For the evaluation of mitochondrial activity, MitoTracker^TM^ Red CMXRos (Molecular Probes, Eugene, OR, USA) was chosen. MitoTracker probes passively diffuse across the plasma membrane and accumulate in actively respiring mitochondria. IPSC were plated on 12 mm diameter round glass coverslips in 24-well plates and then incubated with 150 nM of Mitotracker Red for 30 min. After incubation, the cells were fixed as previously described [15]. Nuclei were stained with the DNA intercalant Hoechst (1:5000). Cells were finally mounted with 50% glycerol in PBS. Confocal analysis was carried out as aforementioned. Fifty random single cells were analyzed per each image using the ImageJ version 1.52t.

### 2.7. Bioinformatics

Gene expression data of undifferentiated T21-iPSCs and controls were retrieved from the Gene Expression Omnibus (GEO) portal. Information on each dataset included in the analysis, with identifiers, platforms and number of cases and controls is reported in Table 1.

Differential expression analysis (DS versus controls) was carried out on individual datasets using the moderated t-statistics implemented in limma [19], or, when RNAseq raw counts were available, by F-tests after fitting a quasi-likelihood negative binomial model with EdgeR [20]. Genes keeping a q-value ≤ 0.05 after multiple-testing correction in the form of the false discovery rate (FDR) were defined as differentially expressed (DEGs).

In order to compare different platforms, all gene identifiers (array-specific probe ID or ENSEMBL gene ID) were harmonized into official HUGO gene symbols using the org.Hs.eg.db annotation package in Bioconductor.

Harmonized datasets were merged with a Z-score based meta-analysis adopting the method implemented in METAL [21]. In brief, this method takes p-values across independent studies as input, with sample size and logFC direction taken into account. First, for each gene, a Z-score is calculated based on the p-values and direction of logFC in each study. Then, the overall Z-score and p-value are calculated from a weighted sum of the individual Z-scores. A gene was defined significantly dysregulated if: (1) holding q ≤ 0.01 after FDR correction; (2) assayed in at least two datasets; (3) non-significant after heterogeneity testing (HetP > 0.05) [22]. The list of dysregulated genes was further analyzed for gene ontology (GO) and pathway enrichment analysis using the Web-based Gene Set Enrichment Analysis Toolkit (WebGestalt) [23] and the combined enrichment score method implemented in EnrichR [24].

### 2.8. [Ca^2+^]_i_ Measurement

[Ca^2+^]_i_ was measured by single cell computer-assisted video-imaging [25]. Briefly, cells grown on glass coverslips were loaded with 10 μM Fura-2 acetoxymethyl ester (Fura-2AM) for 30 min at room temperature in normal Krebs solution containing (in mM) 5.5 KCl, 160 NaCl, 1.2 MgCl_2_, 1.5 CaCl_2_, 10 glucose and 10 Hepes–NaOH, pH 7.4. At the end of the Fura-2AM loading period, the coverslips were placed into a perfusion chamber (Medical System Co., Greenvale, NY, USA) mounted onto a Zeiss Axiovert 200 microscope (Carl Zeiss) equipped with a FLUAR × 40 oil objective lens. Forty to sixty-five individual cells were selected and monitored simultaneously from each coverslip by Me-ta-Morph/MetaFluor Imaging System software (Universal Imaging). Assuming that the KD for FURA-2 was 224 nM, the equation of Grynkiewicz was used for calibration [26]. Then mitochondrial Ca^2+^ extrusion was induced by the treatment with FCCP uncoupler (250 nM). The amount of Ca^2+^ extruded in the cytoplasm upon FCCP exposure, measured as [Ca^2+^]_i_ increase, are widely considered as indexes of mitochondrial Ca^2+^ efflux. All of the results were presented as cytosolic Ca^2+^ concentrations.

### 2.9. Quantification of ATP Content

ATP content was measured by a commercial bioluminescent assay (ATP bioluminescent assay kit, Merck) according to the manufacturer’s instruction and as previously described [27]. Briefly, ATP was extracted by boiling the samples in a solution containing 100 mM TRIS, 4 mM EDTA, pH 7.75. After centrifugation at 10,000× *g* for 60 s, samples were diluted at 1:50 in dilution buffer (FLAA, Merck). To obtain bioluminescence measurements with a standard luminometer, 100 μL of supernatant was mixed with 100 μL of luciferin-luciferase solution. The standard curve of ATP was obtained by serial dilution of 2 μM ATP solution.

### 2.10. Measurement of Reactive Oxygen Species

DCF-DA (2′,7′-Dichlorofluorescin diacetate) (Merck), a cell membrane permeable fluorescein analogue, was used to detect ROS species production [28]. Cells were seeded on glass coverslips. IPSCs and NPCs at day 7 and 21 in the differentiation medium were pre-loaded with DCF-DA (10 µM) for 30 min at 37 °C in PBS. Cells were then washed with PBS, and the reaction was stopped by adding 2,6-di-tert-butyl-4-methylphenol (0.2% in ethanol) and EDTA (2 mM). Each coverslip was placed into a perfusion chamber (Medical System, Co., Greenvale, NY, USA) mounted onto a Zeiss Axiovert 200 microscope (Carl Zeiss, Germany) equipped with MicroMax 512BFT cooled CCD camera (Princeton Instruments, Trenton, NJ, USA). Each coverslip was exposed at 485-nm excitation for 10 s and the emitted light was passed through a 530-nm barrier filter. Image acquisition and processing were performed equally for all experimental conditions; for the quantification, background fluorescence was subtracted from the data.

### 2.11. Mitochondria Bioenergetics Measurements

Real-time measurements of OCR were made using an XFe-96 Extracellular Flux Analyzer (Seahorse Bioscience, Billerica, MA, USA). Cells were plated in XFe-96 plates (Seahorse Bioscience) at the concentration of 30,000 cells/well. Cells were counted before and after the experiments. OCR was measured in XFe media (non-buffered Dulbecco’s modified eagle medium (DMEM) medium containing 10 mM glucose, 2 mM L-glutamine and 1 mM sodium pyruvate) under basal conditions and in response to 2 μM oligomycin, 1.5 μM of carbonyl cyanide-4-(trifluoromethoxy) phenylhydrazone (FCCP) and 1 μM of Antimycin-A and Rotenone (all from Sigma-Aldrich, St. Louis, MO, USA) as previously described [29]. Each sample was plated at least in triplicate. Indices of mitochondrial respiratory function were calculated according to the manufacturer’s instructions.

### 2.12. Statistical Procedures 

Unless otherwise indicated, all assays were performed independently and in triplicate. Statistical analysis was performed using GraphPad Prism software vers.5.0 (GraphPad Software, La Jolla, CA, USA, http://www.graphpad.com, accessed on 2 March 2020). The ANOVA test, with Bonferroni post hoc correction in case of multiple comparisons, was applied to evaluate the statistical significance of differences measured throughout the data sets presented. The threshold for statistical significance (*p*-value) was set at 0.05.

## 3. Results

### 3.1. General Properties of Trisomic and Euploid iPSCs

We preliminarily determined that Eu-iPSCs and T21-iPSCs retained the morphological features previously described [10]. Phase-contrast microscopy images indicated that iPSC clones had the typical morphology of pluripotent stem cells with distinct colony boundaries (Figure S1).

Since human iPSCs are subject to genomic instability [30], we investigated the presence of chromosomal alterations by karyotype analysis according to standard cytogenetic protocols. We confirmed that the Eu-iPSC clone DS2U had a normal male karyotype, whereas the T21-iPSC clones DS1 and DS4 were trisomic for Hsa21 (Figure S2). No other major chromosomal alterations, such as inversions or translocations, were detected by standard karyotyping.

We then determined, by qRT-PCR, the expression levels of stemness (*OCT4* and *NANOG*) and neural differentiation (*PAX6* and *NESTIN*) markers in the iPSCs. Protein expression was evaluated by immunofluorescence and confocal microscopy. The expression of SOX2, known as vital pluripotency factor and as neuroectodermal lineage specifier [31], was also evaluated.

**Figure 2:**
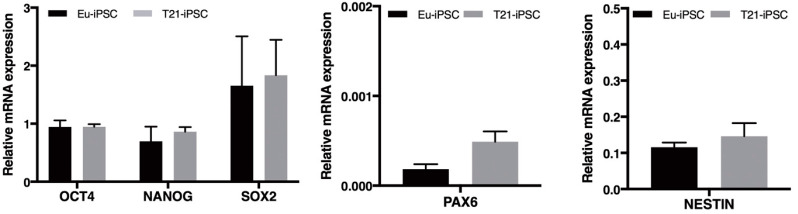
mRNA expression of stemness and differentiation marker genes in euploid and trisomic iPSC clones. Expression of the pluripotency markers, *OCT4*, *NANOG* and *SOX2*, and the neural differentiation markers, *PAX6* and *NESTIN*, was evaluated by qRT-PCR. Pluripotency markers are detected in all iPSCs without significant differences among clones. *NESTIN* and *PAX6* are expressed at very low level in euploid and trisomic iPSCs. mRNA expression levels are normalized to a reference gene (*ACTIN*). Results are expressed as mean values ± SEM of at least four independent experiments for each cell line. T21-iPSC values represent the average of the two trisomic clones used.

All iPSC clones expressed significant levels of the pluripotency markers *OCT4* and *NANOG* (Figure 2). *SOX2* mRNA was expressed at similar levels (Figure 2). Besides, the markers of neural differentiation *PAX6* and *NESTIN* were expressed at very low levels (Figure 2). No difference in the expression level of each marker was observed among the Eu-iPSC and T21-iPSC clones.

**Figure 3:**
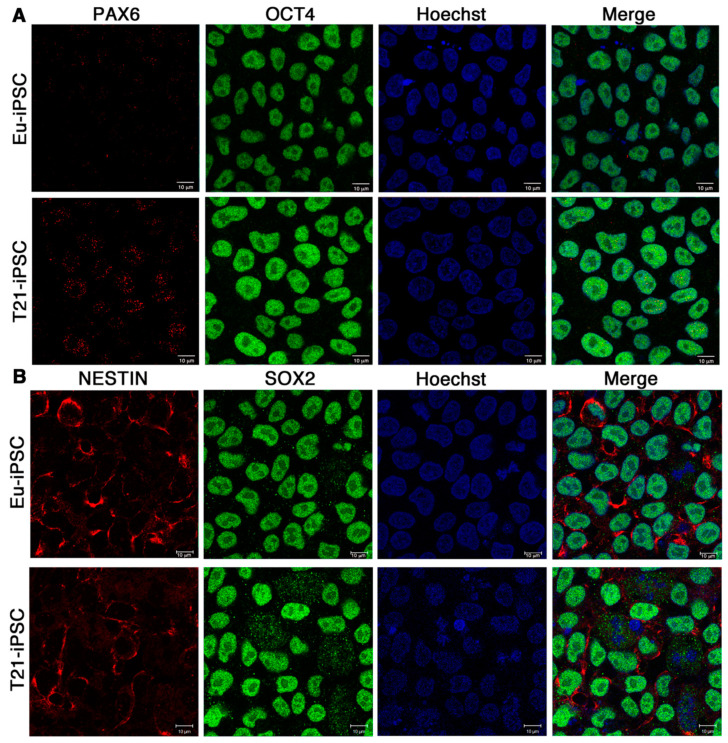
Immunofluorescence detection of stemness and differentiation markers in iPSCs. Expression of OCT4, PAX6, SOX2, and NESTIN was assessed by immunofluorescence and confocal microscopy in both Eu-iPSC and T21-iPSC clones. T21-iPSC images are representative of the two trisomic clones. (**A**) Representative images show that both cell types are negative when stained with the PAX6 antibody (red) and positive with the anti-OCT4 antibody (green). The anti-OCT4 antibody stains the nucleoplasm with the exclusion of nucleoli. (**B**) Both cell types show a low level of signal, mostly in the perinuclear area, when stained with the NESTIN antibody (red). The cell nuclei are positive when cells are stained with the SOX2 antibody (green). Nuclei are also stained with Hoechst (blue). Bar = 10 μm.

Next we assessed the expression of the stemness and neural differentiation markers in iPSCs by immunofluorescence analysis. In full agreement with the qRT-PCR data, we found that Eu-iPSCs and T21-iPSCs resulted positive for the stemness marker OCT4 and negative for the differentiation marker PAX6 (Figure 3A). As expected, the transcription factors PAX6 and OCT4 were localized in the nucleus. Double immunofluorescence analysis allowed to establish that virtually all nuclei were negative for PAX6 and positive for OCT4 (Figure 3A). Eu-iPSCs and T21-iPSCs were similarly positive for SOX2 while they expressed low levels of NESTIN, an intermediate filament protein, which was located in the cytosol and mainly concentrated around the nucleus, as reported for ESCs [32] (Figure 3B).

This preliminary characterization confirmed that the iPSCs retained the morphological features described in previous reports [10,11], had the expected karyotypic set-up, expressed stemness markers and did not express differentiation markers.

### 3.2. Eu-iPSCs and T21-iPSCs Are Efficiently Converted into NPCs in Monolayer Culture by the Dual-SMAD Inhibition Protocol

One of our goals was to develop a model to study the mitochondrial function of iPSCs during their differentiation process into NPCs. Since we thought that the use of high-resolution fluorescence techniques was best suited for our purpose, we used a strategy that would allow us to obtain monolayers of cells without feeder-layers at all steps of the procedure.

The differentiation of euploid and isogenic trisomic iPSCs into neural precursor cells, Eu-NPCs and T21-NPCs respectively, was obtained by the dual-SMAD inhibition protocol [9] in monolayer cultures (see Matherial and Methods). This technique demonstrated a high efficiency and promoted rapid differentiation of iPSCs into NPCs. Moreover, by avoiding the passage of neurosphere formation, the technique resulted less operator-dependent and hence highly reproducible.

**Figure 4:**
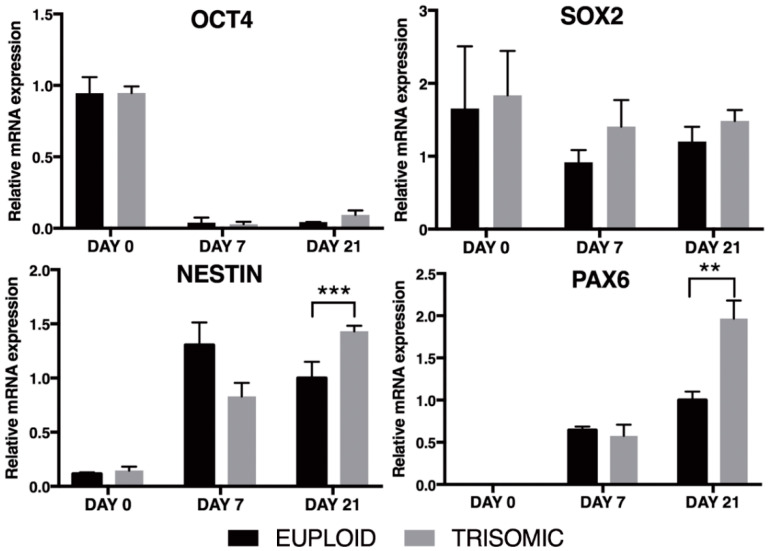
qRT-PCR analysis of stemness and neural differentiation markers. The stemness, *OCT4*, and neural differentiation markers, *NESTIN*, *SOX2,* and *PAX6*, were analyzed by qRT-PCR in euploid and trisomic cells, at different times of differentiation in culture. mRNA expression is normalized to a reference gene (*ACTIN*). Results are expressed as mean values ± SEM of at least four independent experiments for each cell line. ** *p* < 0.01; *** *p* < 0.001. Values of trisomic cells represent the average of the two trisomic clones used.

To monitor the differentiation process we assessed, by qRT-PCR, the expression level of the same stemness and neural differentiation markers previously described. A severe reduction in the expression of the stemness marker *OCT4* was observed already at day 7 and maintained up to day 21 of culture (Figure 4). Concomitantly, a time-dependent increase in the expression of *NESTIN* and *PAX6*, with a more significant increase in trisomic clones cultured for 21 days, was found. *SOX2* expression did not change significantly during the differentiation period (Figure 4), in agreement with its role in maintaining neural progenitor stem cell properties [31].

**Figure 5:**
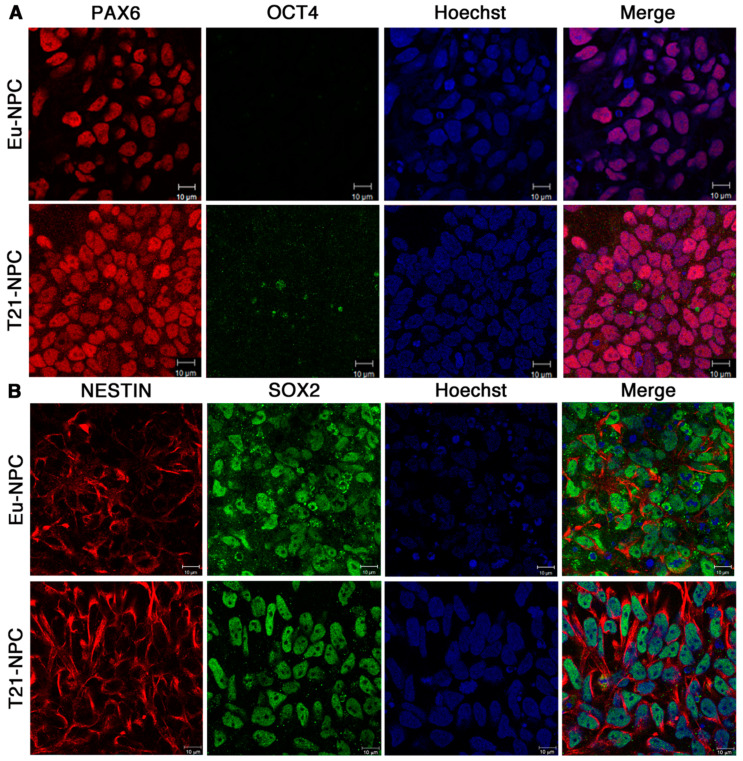
Expression of stemness and differentiation markers in iPSC-derived cells after seven days of neural induction. Expression of OCT4, PAX6, SOX2, and NESTIN was assessed by immunofluorescence in iPSCs after seven days of culture in differentiation medium. T21-NPC images are representative of the two trisomic clones. (**A**) Both euploid and trisomic cells are negative for OCT4 (green) and positive for PAX6 (red) staining. (**B**) Both cell types are positive when stained with NESTIN and SOX2 antibodies. The NESTIN antibody stains the intermediate filaments in the cytoplasm (red) while the SOX2 antibody stains this transcription factor in the nucleus (green). Nuclei are also stained with Hoechst (blue). Bar = 10 μm.

Consistently, immunofluorescence analyses revealed similar behavior of trisomic and euploid cells at the protein level. Both NPC lines had positive nuclei when stained with the anti-PAX6 antibody and negative nuclei when stained with the anti-OCT4 antibody (Figure 5A). Considering that we used a double immunofluorescence technique, it was possible to assess that PAX6-positive nuclei were also OCT4-negative. Since nuclei were also stained by Hoechst, we were able to evaluate that the differentiation efficiency was higher than 90% in different experiments.

In a separate set of experiments, we found that full expression of the PAX6 marker already occurred by day 5 of culture in differentiation medium in agreement with many studies reporting that PAX6 is the earliest human neuroectoderm cell fate determinant [33,34]. Loss of expression of the OCT4 marker also occurred within the same time frame (Figure S3).

Two other neural differentiation markers, SOX2 and NESTIN, were also expressed in euploid and trisomic cells at day 7 of culture. Expression levels were high and easily detectable by immunofluorescence analysis (Figure 5B). The very high percentage of positive cells was similar to that observed for PAX6, and again no significant difference was observed between euploid and trisomic cells.

**Figure 6:**
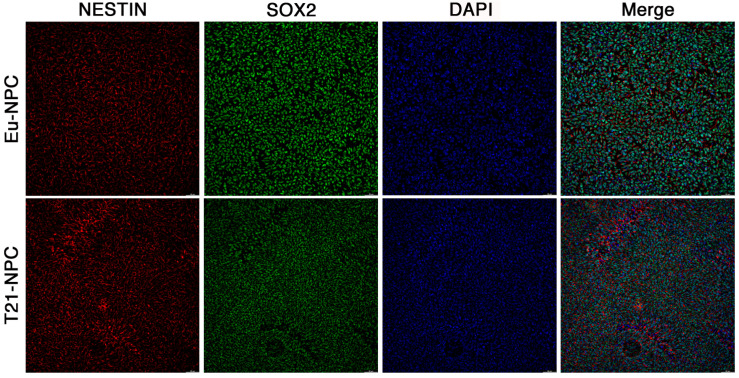
Low magnification acquisition of NESTIN and SOX2 positive NPCs. Euploid and trisomic NPCs were stained by double immunofluorescence with anti-NESTIN and anti-SOX2 antibodies at seven days of culture in differentiation medium. Fluorescent images were taken by the Thunder Imaging Systems. T21-NPC images are representative of the two trisomic clones. Eu-NPCs and T21-NPCs are positive when stained with either the NESTIN antibody (red) or the SOX2 antibody (green). Nuclei are also stained with DAPI (blue). Note the uniform staining of NESTIN and SOX2. Bar = 50 μm.

To further prove that the vast majority of cells were positive for both SOX2 and NESTIN in the double labeling experiments and to assess the uniformity of cell monolayers, we acquired images of the cultures at low magnification. To this aim, we used the THUNDER Imaging Systems, which allowed to inspect large areas of the cell monolayer with high quality and at high speed. In full agreement with the results of higher resolution pictures, the data indicated that the vast majority of cells were positive for both markers (Figure 6).

In summary, by day 7 of induction, or even earlier, Eu-iPSCs and T21-iPSCs lose stemness markers and acquire NPCs differentiation properties. The cell culture strategy used is simple and promotes the formation of NPC monolayers within days and with very high differentiation efficiency.

### 3.3. Prolonged Induction of NPCs Unveals a Glial-Type Differentiation Potential of Trisomic Cells

**Figure 7:**
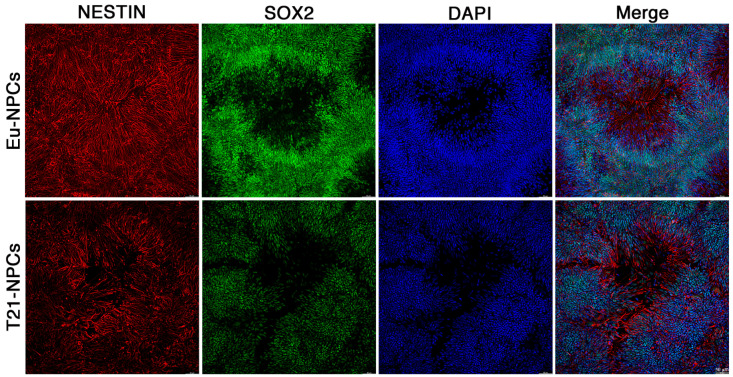
Expression of NESTIN and SOX2 in Eu-NPCs and T21-NPCs at day 21 of culture. Immunofluorescence detection of NESTIN (red), SOX2 (green) and DAPI (blue) at day 21 of treatment with differentiation medium. T21-NPC images are representative of the two trisomic clones. The nuclei, stained in green or blue, are clustered in some areas. NESTIN filamentous pattern is clearly detected in areas without nuclei. Pictures were taken at the THUNDER Imaging Systems. Bar = 50 μm.

NPCs were cultured in differentiation medium until day 21. Both euploid and trisomic cells were still positive for all differentiation markers, with higher expression of *NESTIN* and *PAX6* in the trisomic cell cultures as detected by qRT-PCR (Figure 4). At day 21, a dramatic change in the organization and distribution of NESTIN was observed by immunofluorescence staining. While nuclei appeared clustered, the NESTIN signal often appeared as filamentous structures that extended into areas lacking nuclei (Figure 7). This distribution of NESTIN was consistent with that expected from neuronal differentiation with the formation of cytoplasmic protrusions that will become either axons or dendrites (Figure 7).

**Figure 8:**
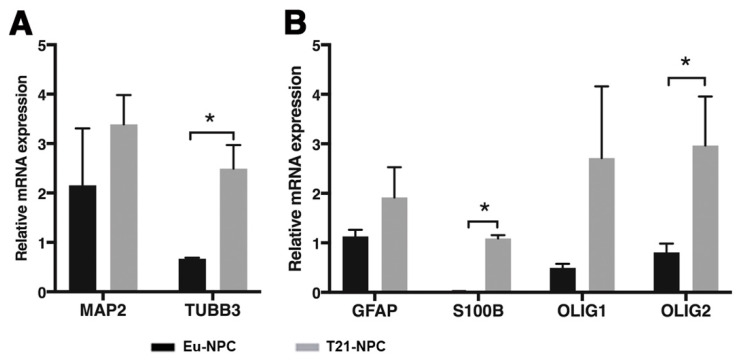
Quantitative qRT-PCR analysis of neuronal, astroglial and oligodendroglial markers in NPCs at day 21 of neural induction. (**A**) The differentiated neuronal cell markers, *MAP2* and *TUBB3*, were analyzed by qRT-PCR in euploid and trisomic NPCs, at day 21 of neural induction. An higher level of *TUBB3* expression is observed in T21-NPCs. (**B**) The level of expression of *GFAP*, *S100B*, *OLIG1* and *OLIG2*, was analyzed by qRT-PCR in euploid and trisomic NPCs. mRNA expression is normalized to a reference gene (*ACTIN*). T21-NPC values represent the average of the two trisomic clones used. Results are expressed as mean values ± SEM of at least four independent experiments for each cell line. * *p* < 0.05.

To assess whether the NPCs were undergoing neuronal differentiation, the expression level of two specific markers, *MAP2* and *TUBB3*, was determined by qRT-PCR. Both neuronal markers were expressed on day 21 of culture, with a significantly higher level of *TUBB3* in T21-NPCs compared with Eu-NPCs (Figure 8A), suggesting an altered differentiation process of T21-NPCs.

It is well established that the brain formation process in DS, in addition to impaired neurogenesis, is characterized by a prominent representation of glial cells [35]. Thus, we asked whether glial cells formed preferentially in trisomic versus euploid cell cultures by measuring the expression level of a set of glial markers (*GFAP*, *S100B*, *OLIG1*, and *OLIG2*) in Eu-NPCs and T21-NPCs. Note that *OLIG1*, *OLIG2*, and *S100B* genes map to Hsa21 and may be overexpressed due to dosage effect. qRT-PCR revealed a higher expression level of all these genes in T21-NPCs compared with Eu-NPCs at day 21 of culture, with significantly higher levels of *S100B* and *OLIG2* (Figure 8B).

Taken together, these data indicate that NPCs are undergoing neuronal differentiation by day 21 in the induction medium, and suggest that trisomic NPCs may acquire a more glial phenotype following prolonged culture.

### 3.4. Mitochondria-Related Gene Expression Is Dysregulated in iPSCs

In order to investigate the expression profile of mitochondria-related genes and dysregulated pathways in iPSCs derived from DS subjects, we performed a meta-analysis of gene expression data.

The specific purpose of this analysis was to investigate whether human iPSCs with chromosome 21 trisomy exhibit any perturbation in pathways and GO categories that affect mitochondrial function and/or morphogenesis.

Four gene expression datasets of undifferentiated T21-iPSCs and controls were retrieved from Gene Expression Omnibus (GSE48611 [10], GSE52249 [16], GSE42956 [17] and GSE101942 [18]), which include a total of 49 biological samples (24 trisomic iPSCs and 25 euploid iPSCs) from three different platforms (Table 1). Each experiment was first analyzed independently (see Materials and Methods) and then the data merged in a meta-analysis based on the *p*-values obtained in the individual experiments. We found 295, 3354, 2718, and 152 genes dysregulated in the GSE48611, GSE52249, GSE42956, and GSE101942 datasets, respectively. A total of 28,675 genes entered the Z-score based meta-analysis and 2474 of them were found significantly dysregulated: 870 were upregulated and 1604 were downregulated. As a proof of concept, we conducted a gene set enrichment analysis on these genes using the chromosome positional gene set collection and, as expected, we found a significant enrichment of genes mapping to Hsa21 in the list of genes upregulated in T21-iPSCs (*p* = 4.81 × 10^−16^) (Table S1).

Notably, WebGestalt functional analysis of GO terms for cellular component of genes differentially expressed in T21-iPSCs showed that the “mitochondrion” term (GO:0005739) was highly enriched (FDR = 5.1 × 10^−9^) with 213 genes observed in the list, 155 downregulated and 58 upregulated, instead of the 139 genes expected by chance (Table 2). Classification of these 213 dysregulated genes, according to the GO Biological Process categories, highlighted that most of them were involved in mitochondrion organization, calcium homeostasis, mitochondrial fission and fusion, and autophagic processes (Table S2).

Downregulated mitochondria-related genes were significantly associated to the GO:0032592 category ‘Integral component of mitochondrial membrane’, which includes TOMM20, IMMT, and two genes involved in the mitochondrial calcium uptake, namely MCUR1 and MICU1 (Table S3). Notably, the mitochondrial calcium uniporter gene MCU was significantly upregulated in T21-iPSCs.

When genes upregulated in T21-iPSCs versus euploid controls were classified by EnrichR according to pathway analysis using the Molecular Signatures Database v7.4 (MSigDB—Collection: hallmark gene sets 2020) [36,37], the ‘mammalian target of rapamycin complex 1 (mTORC1) signaling’ was the most significantly enriched pathway with 37 dysregulated genes out of the 200 genes of the pathway (Table S4). The signaling pathway activated by mTORC1 regulates at multiple levels mitochondrial function [38].

This analysis indicates that the expression profile of mitochondria-related genes is significantly altered in iPSCs derived from DS subjects compared with euploid controls. Dysregulation of these genes is likely to affect the mitochondrial phenotype in T21-iPSCs.

### 3.5. Mitochondrial Functional Differences between Trisomic and Euploid Cells Manifest at an Early Stage of Neuronal Differentiation

**Figure 9:**
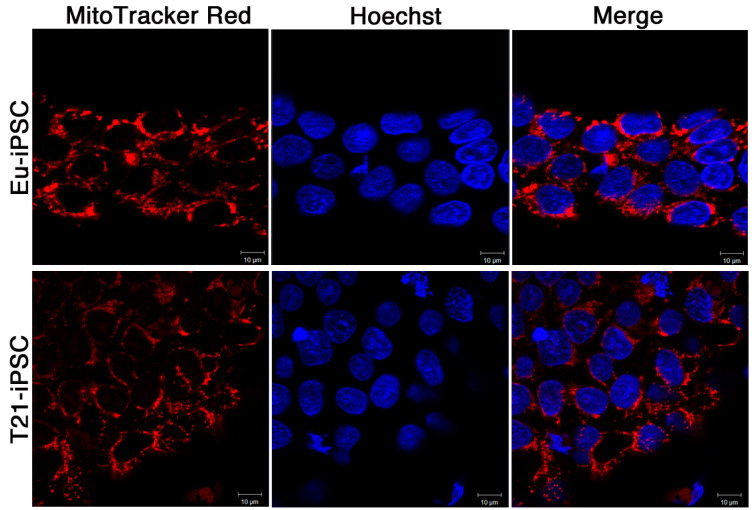
Organization of the mitochondrial network in euploid and trisomic iPSCs. Confocal microscopy representative images of the MitoTracker Red-related fluorescence of euploid and trisomic iPSC clones. MitoTrackerRed probe stains mitochondria (red). Nuclei are stained with Hoechst (blue). Bar = 10 μm.

We investigated mitochondrial network organization in iPSCs by immunofluorescence using the MitoTracker Red dye, a reagent that stains mitochondria in live cells. Morphological analysis indicated that mitochondria of iPSC clones had a spherical, condensed, and immature-like structure, as expected from pluripotent stem cells. Mitochondria showed densely packed perinuclear localization and a poorly organized filamentous network structure (Figure 9) in agreement with previous reports [39].

Overall, no significant change in mitochondrial network organization could be demonstrated in T21-iPSCs compared with Eu-iPSCs. 

**Figure 10:**
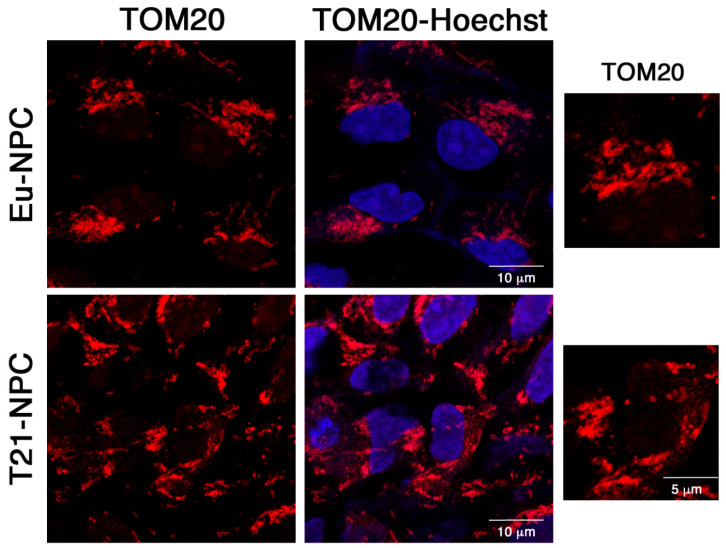
Organization of the mitochondrial network in euploid and trisomic NPCs at day 7 of culture in differentiation medium. Confocal microscopy representative images of the TOM20-related fluorescence of euploid and trisomic NPC clones at day 7 of neural induction. TOM20 antibody (in red) stains the outer mitochondrial membrane. Nuclei are stained with Hoechst (blue). Bar = 10 μm. Higher magnification area from the same picture are shown on the right. Bar = 5 μm.

We therefore wondered whether the mitochondrial network underwent any changes during neural induction. We analyzed by immunofluorescence the mitochondrial distribution of the outer mitochondrial membrane protein TOM20 in euploid and trisomic NPCs at day 7 of induction. We observed that the mitochondrial network acquired a more organized aspect, regardless of Hsa21 trisomy (Figure 10).

We therefore wondered whether the mitochondrial network underwent any changes during neural induction by labeling mitochondria with an antibody against the outer mitochondrial membrane protein TOM20 in euploid and trisomic NPCs at day 7 of induction (Figure 10). Interestingly, in the transition from iPSCs to NPCs, we observed a mitochondrial redistribution within the cells. Indeed, while in iPSCs mitochondria were mainly perinuclear, in euploid and trisomic NPCs, they displayed a more widespread distribution as evident in higher magnification pictures (Figure 10).

**Figure 11:**
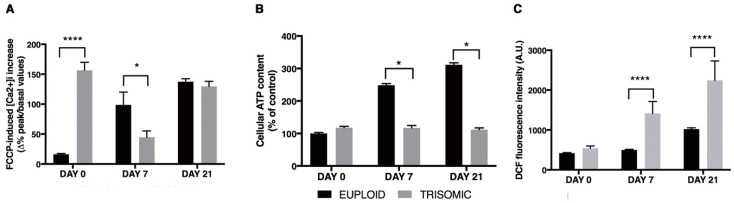
Analysis of mitochondrial function in euploid and trisomic iPSCs and NPCs at day 7 and 21 of culture in differentiation medium. (**A**) The level of [Ca^2+^]_i_ was measured with Fura-2AM after FCCP treatment. The bars show the mean values ± SEM of determinations, as percentage difference of peak values (after FCCP) with respect to basal [Ca^2+^]_i_, from at least 25 cells/condition. (**B**) Cellular ATP content, measured by an ATP bioluminescent assay, was expressed as percentage of Eu-iPSC values (set equal to 100). The bars show the mean values ± SEM of determinations from three independent experiments. ATP levels were normalized to protein content of each sample. (**C**) ROS production was measured by DCF fluorescence intensity. The bars show the mean values ± SEM of determinations, in arbitrary units, from at least 25 cells/condition. Values of trisomic cells represent the average of the two trisomic clones used. * *p* < 0.05; **** *p* < 0.0001.

In view of the central role of mitochondria in maintaining cellular Ca^2+^ homeostasis and Ca^2+^ signaling, we determined Ca^2+^ content in Eu-iPSCs and T21-iPSCs, and in derived NPCs. We performed single-cell analysis of the mitochondrial Ca^2+^ signaling pathway in iPSCs (day 0) and NPCs at day 7 and 21 (Figure 11A). At day 0, FCCP treatment led to a rapid increase in the intracellular Ca^2+^ concentration ([Ca^2+^]_i_), which was significantly higher in T21-iPSCs than in the Eu-iPSCs. Then, it decreased in T21-NPCs at day 7 becoming higher at day 21. On the other hand, FCCP-induced Ca^2+^ release progressively increased in Eu-NPCs at day 7, reaching levels similar to that of T21-NPCs at day 21 (Figure 11A).

Energy status in living cells is determined by ATP nucleotide levels, which are mainly dependent on mitochondrial activity [40]. Loss of mitochondrial function in terms of ATP generation has been documented in cultured human DS fetal fibroblasts [29,40,41]. Therefore, ATP content levels were measured in euploid and trisomic iPSCs and NPCs. ATP content increased significantly during physiological differentiation of Eu-NPCs, peaking at day 21. No increase was instead detected in T21-NPCs over time in culture (Figure 11B). 

Mitochondria play a key role in oxygen metabolism and they are an important source of ROS formation. ROS hyperproduction was found in cellular models of DS [42,43,44] likely as a result of mitochondrial dysfunction. ROS production, monitored by DCF, progressively increased in T21-NPCs at day 7 and 21, and it was significantly higher than in Eu-NPC cultures (Figure 11C).

**Figure 12:**
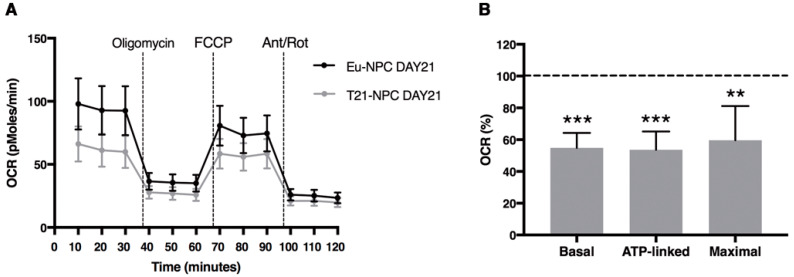
Mitochondrial respiratory function in euploid and trisomic NPCs at day 21 of culture in differentiation medium. (**A**) Merged curves of mean values of OCR obtained in basal condition and after consecutive addition of oligomycin, FCCP and Antimycin-A/Rotenon. (**B**) Relative mean values ± SEM of Basal OCR, ATP-linked and maximal respiration of two T21-NPC clones were expressed as percentage of Eu-NPC values, set equal to 100 (dashed line). Each experiment was carried out at least in triplicate. ** *p* < 0.01; *** *p* ≤ 0.001.

All together, these data are strongly suggestive of an overall altered function of mitochondria in trisomic cells, which essentially appears during neuronal differentiation as early as day 7 of neural induction. As confirmation of these alterations in mitochondrial bioenergetics, we directly measured Basal OCR, ATP-linked respiration, and maximal respiration by the XFe-96 Extracellular Flux Analyzer. T21-NPCs at day 21 showed significant lower values for all parameters when compared with Eu-NPCs (Figure 12). However, the increase in OCR after addition of FCCP did not reach greater values than those observed at baseline, thus resulting in a very low respiratory capacity, regardless of the ploidy of the cells.

## 4. Discussion

We have established a protocol for obtaining monolayer cultures of T21-iPSCs that differentiate into T21-NPCs. We found that T21-NPCs manifest a glial phenotype not present in controls, and simultaneously show clear alterations in mitochondrial function. We discuss here: (i) the advantages of using iPSCs to model defects in neurogenesis; (ii) the early acquisition of a gliocentric differentiation phenotype by T21-NPCs; (iii) the mitochondrial dysfunction whose molecular basis is already present in T21-iPSCs but manifests more strikingly in T21-NPCs.

### 4.1. T21-iPSCs to Model Defects in Neurogenesis

Disease-specific iPSCs are a valuable tool for in vitro modeling of neurodevelopmental disorders and other complex human diseases [45]. T21-iPSCs have already been used to model neural development in DS. The main anomalies observed in neural population derived from trisomic iPSCs were neurogenesis impairment and oxidative stress [10,16,17,46]. NPCs and neurons from T21-iPSCs resemble gene expression patterns from fetal DS tissue [10,47], and appear quite similar to the prenatal brain; they may help in understanding key aspects of prenatal human brain development that are not accessible by other means [45]. Although previous experiments have thoroughly investigated many features of the neural population derived from T21-iPSCs, mitochondrial function has been poorly evaluated in these cells. The present study aimed to fill this gap by investigating the efficiency of the mitochondrial bioenergetics during the early stages of neuronal differentiation. We have adopted a protocol in which cells are grown in monolayers without feeder-layers and differentiate in 2D culture conditions, by the dual-SMAD inhibition protocol, without embryo bodies or neural rosettes selection. The novelty of our procedure is that we used a combination of technical approaches that allowed us to develop an easy, efficient, fast, and reproducible protocol. This choice enabled confocal microscopy examination and single cell studies of functional parameters with high-resolution fluorescence techniques, which are very convenient for the study of mitochondrial function, at different time points. We are aware that by using 2D monolayer cultures, we may lose some of the advantages of 3D organoid cultures [48] although a direct comparison of 2D vs 3D neural induction methods demonstrated that both are equally suitable for generating different classes of neurons from human iPSCs [49].

A preliminary characterization of the cells used in this study demonstrated that all iPSCs retained the morphological characteristics described in previous reports [10], so we proceeded to evaluate their properties during neural differentiation. After seven days of induction, both Eu-iPSCs and T21-iPSCs lost the stemness marker OCT4 and acquired the differentiation properties of NPCs becoming positive for PAX6 and NESTIN markers. Indeed, a full expression of the PAX6 marker was already visible by day 5 of culture in differentiation medium along with a loss of OCT4 marker expression. THUNDER microscopy imaging indicated that the vast majority of cells were positive for the neuronal differentiation marker NESTIN as well as the pluripotency and neural differentiation marker SOX2 [31,50], in full agreement with the results of higher magnification analyses. This data further supports the efficacy of the dual-SMAD inhibition/2D differentiation protocol [9] that is highly reproducible and does not require to pick rosettes or to obtain embryo-bodies and neurospheres, whose fluorescence analysis is more complex and high resolution is difficult to be achieved.

### 4.2. Gliocentric Differentiation Phenotype

No significant differences were observed between euploid and trisomic cells at day 7 of induction with regard to the expression of stemness and differentiation markers. However, at day 21 of culture, trisomic cells exhibited a higher level of *NESTIN* expression. The protein was frequently distributed, in both trisomic cells and controls, in cellular extensions likely in the process of forming neurites. By the same time, we also observed that several glial markers were overexpressed in T21-NPCs. In particular, *S100B* and *OLIG2*, which map to Hsa21, showed much higher expression levels in T21-NPCs than expected from the gene dosage effect alone. This is strongly suggestive of the emergence in trisomic cultures of a glial-related cell population, likely astroglia. It is well established that the brain of DS individuals is characterized by hypotrophy and hypocellularity of neurons [35,51] and oligodendrocytes [52], whereas astroglial cell numbers are increased [53,54]. A substantial increase in the number of glial fibrillary acid protein (GFAP) positive cells, both astrocytes and radial glial cells, was found in DS fetuses at 18–20 weeks of gestation [53], indicating that the unbalance between neurons and glial cells is an early event during fetal development. The occurrence of a gliogenic shift in neural population derived from trisomic iPSCs has been reported by several authors, although there has been disagreement on the specific populations and timing of appearance of glial cells [10,16,17]. Our results support the notion that T21-NPCs acquire a more glial phenotype as early as 21 days from the onset of iPSC neural induction. 

Differentiating iPSCs provided some insight into the mechanisms underlying the appearance of the gliogenic phenotype. The increased expression of the glial markers S100B, OLIG1, OLIG2, GFAP, CNPase, VIM, O4 and PDGFRA, has been taken into account [46,55,56]. A cross-talk between JAK/STAT and NOTCH signaling pathways is crucial for the promotion of astrocytic differentiation of NPCs [57]. As a consequence of the overexpression of triplicated genes, the activity of the JAK-STAT pathway is increased, leading to overexpression of *GFAP* and *S100B*, thus promoting the enhancement of astrogliogenesis [35] even though other Hsa21 genes, such as *C21orf91*, may contribute to this event as well [58]. We provide evidence here that, by day 21, a significant increment of *S100B* and *OLIG2* mRNA occurs, which might represent a very early event in the gliogenic process.

### 4.3. Mitochondrial Dysfunction in T21-iPSCs and T21-NPCs

A major aim of our study was to investigate the mitochondrial function at early steps of neuronal differentiation. Transcriptome studies in DS fetal tissues demonstrated that nuclear encoded mitochondrial genes represent a major downregulated category [44,59,60]. Genes and miRNAs located on Hsa21 could influence mitochondrial function by targeting genes belonging to this category [61,62,63,64]. In agreement with these findings, it has been demonstrated that T21 decreased the efficiency of the mitochondrial energy production apparatus, disrupting mitochondrial morphology, and increasing mtCa^2+^ load and ROS production in human primary trisomic fibroblasts [41,43,44,65].

Despite this extensive literature, little information is available about the mitochondrial phenotype in the developing brain of human DS subjects. Main questions are: at what stage of neuronal differentiation does mitochondrial dysfunction occur? Is the basis for the dysfunction already present in trisomic stem cells? To answer these questions, we investigated the transcriptome features of mitochondria-related pathways in trisomic iPSCs. To this end, we reanalyzed public gene expression data, including those obtained from the samples we used in our experiments. Gene set enrichment analysis of genes dysregulated between trisomic and euploid iPSCs revealed that the “mitochondrion” GO term for Cellular Component was significantly over-represented. Most of these genes were involved in mitochondrion organization, calcium homeostasis, mitochondrial fission and fusion and autophagic processes. About this last category, EnrichR pathway analysis of DEGs upregulated in T21-iPSCs indicated mTORC1 signaling as the most significantly enriched pathway, as about 20% of genes of the pathway were upregulated. The list included a number of genes involved in the regulation of the autophagic process [66]. The overexpression of these genes, especially that of the autophagy adaptor SQSTM1/p62, an oxidative stress inducible gene, may negatively regulate autophagy by activating mTORC1 [67,68], a complex that regulates protein synthesis, autophagy, mitochondrial function and mitophagy [38,68]. This last role is further supported by the demonstration that mitophagy is induced following treatment with mTORC1 inhibitors [69]. In summary, the meta-analysis demonstrated that the molecular bases of mitochondrial dysfunction are already present in iPSCs derived from DS subjects, which manifest global dysregulation of mitochondria-related genes. This condition is likely to affect mitochondrial function, morphology, and network in these cells.

On these premises, we investigated mitochondrial organization in euploid and trisomic iPSCs. Both showed mitochondria with an immature-like structure, a densely packed perinuclear localization, and a poorly organized filamentous network structure. It is known that during reprogramming into iPSCs mitochondria revert to an immature state in terms of organelle morphology and distribution, expression of nuclear factors involved in mitochondrial biogenesis, and intracellular ATP level [70]. Human iPSCs, like ESCs, generally exhibit few mitochondria with underdeveloped cristae, with the exception of cells at the periphery of the colonies, which may be in the process of differentiating [71]. During differentiation, mitochondria become mature and functionally active, and the energy metabolism switches from anaerobic glycolysis to oxidative phosphorylation [72,73]. In agreement with these notions, we did not observe significant differences in euploid and trisomic undifferentiated cells with respect to ATP and ROS production. This is not surprising because pluripotent stem cells, which rely more on anaerobic rather than aerobic mitochondrial respiration, produce low levels of intracellular ATP and ROS [72,74,75]. However, by day 7, ATP content progressively increased in euploid cells while it remained unchanged in trisomic ones. On the contrary, ROS production dramatically increased in T21-NPCs over time. Mitochondrial bioenergetics was affected accordingly, as demonstrated by the significant decrease of Basal OCR, ATP-linked respiration, and maximal respiration in T21-NPCs at day 21 when compared with Eu-NPCs. These results indicate that mitochondrial activity is altered at an early stage during neuronal differentiation of T21-iPSCs as a possible consequence of the dysregulation of those Hsa21 genes that are known to play a role in the control of mitochondrial function [65].

Interesting inferences were drawn from the study of calcium during neural differentiation of T21-iPSCs. The intracellular Ca^2+^ concentration demonstrated an oscillating trend in these cells after treatment with an uncoupler of oxidative phosphorylation. In the undifferentiated state, the concentration dramatically increased in trisomic cells, more than in euploid ones. Ca^2+^ concentration progressively increased in Eu-NPCs at day 7 and 21, possibly due to their organellar competence acquisition. Conversely, Ca^2+^ concentration decreased in trisomic cells at day 7 to increase again at day 21. Deregulation of Ca^2+^ homeostasis and Ca^2+^-mediated signaling has already been described in cells derived from trisomic patients and in murine models of DS [44,76,77]; however, here we have identified the earliest time frame during in vitro neural differentiation in which these events originate. We formulate the hypothesis that the mitochondrial Ca^2+^ signaling pathways during differentiation might be compromised in T21-NPCs because of the abnormal peak of mitochondrial Ca^2+^ content in T21-iPSCs. This condition, together with overexpression of the calcium-binding protein S100B, which is known to be able to promote a gliocentric shift in DS neural progenitors [55,56,78], could be in part responsible for the increased expression of neuronal and glial markers in T21-NPCs. The increase in intracellular Ca^2+^ concentration has been suggested to be the core signal that controls neural fate in vertebrates [79,80]. Spontaneous Ca^2+^ fluctuations, which drive suppression of global excitability in co-cultured neurons, were also observed in DS-derived astroglia [12].

Movement of Ca^2+^ across the inner mitochondrial membrane requires the mitochondrial calcium uniporter (MCU), which modulates mitochondrial Ca^2+^ uptake when the concentration of this ion is elevated in the cytosol [81]. The sigmoidal response of the organelle Ca^2+^ uptake pathway is not intrinsic to the channel itself, but rather is provided by the interaction with the MICU1 complex. As soon as extramitochondrial [Ca^2+^] increases, Ca^2+^-dependent MICU1 activation ensures the prompt initiation of rapid mitochondrial Ca^2+^ accumulation [82]. The meta-analysis we performed on T21-iPSC public expression data demonstrated that MICU1 was downregulated, whereas MCU was upregulated in these cells, which is in agreement with the [Ca^2+^] accumulation in mitochondria and, consequently, the rapid increase in its intracellular concentration after FCCP treatment of T21-iPSCs. Finally, mitochondrial Ca^2+^ overload may be an important cause of mitochondrial ROS generation [83,84], although a large part of the ROS increase could be produced by glial cells and it could also result from the malfunction of the mitochondrial respiratory chain.

## 5. Conclusions

We show here that it is easy to obtain from trisomic iPSCs neural precursors that form monolayers and manifest alterations, such as preferential growth of the glial component, as early as day 21 of culture. This cell model can be conveniently used to monitor, and possibly counteract with drug treatments, neurogenesis defects. The development of assays for high-throughput screening of active drugs appears therefore feasible.

The most important result we obtained using this experimental model is that mitochondrial dysfunction occurs early in NPCs. We identified the earliest time frame in which mitochondrial dysfunction manifests during neural differentiation. Since recent evidence suggests that mitochondria are central regulators of neural stem cell fate decisions during development [85], it seems plausible to hypothesize that mitochondrial defect may play a role in the alterations of neurogenesis that have been observed in differentiating trisomic stem cells. This hypothesis can now be tested as it has already been shown that the mitochondrial defects in trisomic cells can be corrected by the use of common drugs such as metformin [29] and pioglitazone [65].

Overall, these results represent further advancement to lay the foundation for testing therapeutic approaches to counteract the cognitive defect in DS.

### Limitations of This Study

A limitation of this study lies in the fact that, to take advantage of the use of isogenic cells, we considered only a limited number of samples. In addition, this work shares with many others the limitation of being an in vitro cell culture study. We do not know, therefore, to what extent it reflects the timing and sequence of molecular differentiation events that occur in vivo.

## Figures and Tables

**Table 1:** Datasets included in the gene expression meta-analysis of trisomic (DS) and euploid (Controls) iPSCs.

GSE ID	Type	Platform	Genes *	DS **	Controls **
GSE48611 [10]	microarray	Affymetrix HG U133 Plus 2.0	20,534	6	3
GSE52249 [16]	RNAseq	Illumina HiSeq 2000	16,214	3	4
GSE42956 [17]	microarray	Illumina Human HT-12V4.0	21,037	12	15
GSE101942 [18]	RNAseq	Illumina HiSeq 2000	17,251	3	3

* Number of genes included in the meta-analysis, ** Number of DS and Control samples.

**Table 2:** The top 12 cellular component GO terms enriched of DEGs in T21-iPSCs from Webgestalt analysis.

Gene Set	Description	Size	Expected	Observed	Ratio	*p* Value	Adjusted *p* Value
GO:0015630	microtubule cytoskeleton	1164	103.88	186	1.79	7.7716 × 10^−16^	9.1316 × 10^−13^
GO:0005694	chromosome	1014	90.49	165	1.82	7.7716 × 10^−15^	4.5658 × 10^−12^
GO:0031984	organelle subcompartment	1661	148.23	237	1.61	1.9984 × 10^−14^	7.8271 × 10^−12^
GO:0044427	chromosomal part	886	79.07	146	1.85	1.1569 × 10^−13^	3.3983 × 10^−11^
GO:1902494	catalytic complex	1346	120.12	196	1.63	1.3223 × 10^−12^	3.0482 × 10^−10^
GO:1990234	transferase complex	766	68.36	128	1.87	1.5565 × 10^−12^	3.0482 × 10^−10^
GO:0005794	Golgi apparatus	1516	135.29	214	1.58	2.3663 × 10^−12^	3.9721 × 10^−10^
GO:0031967	organelle envelope	1156	103.16	172	1.67	6.2294 × 10^−12^	8.1328 × 10^−10^
GO:0031975	envelope	1156	103.16	172	1.67	6.2294 × 10^−12^	8.1328 × 10^−10^
GO:0044430	cytoskeletal part	1620	144.57	221	1.53	2.8619 × 10^−11^	3.3627 × 10^−9^
**GO:0005739**	**mitochondrion**	**1555**	**138.77**	**213**	**1.53**	**4.7783** × 10^−11^	**5.1041** × 10^−9^
GO:0005730	nucleolus	939	83.80	143	1.71	8.5312 × 10^−11^	8.3535 × 10^−9^

The GO term “mitochondrion” was very significantly enriched (FDR = 5.1041 × 10^−9^) with 213 genes observed in the list instead of the 139 genes expected by chance. The bold is to highlight the mitochondrion category.

## Data Availability

GEO Datasets analyzed in this study: GSE48611 https://www.ncbi.nlm.nih.gov/geo/query/acc.cgi?acc=GSE48611; GSE52249 https://www.ncbi.nlm.nih.gov/geo/query/acc.cgi?acc=GSE52249; GSE42956 https://www.ncbi.nlm.nih.gov/geo/query/acc.cgi?acc=GSE42956; GSE101942 https://www.ncbi.nlm.nih.gov/geo/query/acc.cgi?acc=GSE101942.

## Supplementary Materials

The following are available online at https://www.mdpi.com/article/10.3390/biology10070609/s1. Table S1. GO Biological Process categories of mitochondria related genes dysregulated in iPSCs derived from DS subjects. The most interesting GO terms are highlighted in bold; Table S2: GO cellular component category for ‘Integral component of mitochondrial membrane’ of DEGs downregulated in T21-iPSCs; Table S3: GO cellular component category for ‘Integral component of mitochondrial membrane’ of DEGs downregulated in T21-iPSCs; Table S4. mTORC1 signaling enriched of genes upregulated in T21-iPSC from EnrichR analysis; Figure S1. Morphological characteristics of iPSCs; Figure S2. Karyotype of Eu-iPSC and T21-iPSC clones; Figure S3. Expression of stemness and differentiation markers in iPSC-derived cells after 5 days of neural induction.
