# Supplementary material for: Human Trisomic iPSCs from Down Syndrome Fibroblasts Manifest Mitochondrial Alterations Early during Neuronal Differentiation
Source: Biology (Basel). 2021 Jun 30;10(7):609. doi: 10.3390/biology10070609 (PMC8301075; doi:10.3390/biology10070609)
Supplement: Supplementary file 1 [file biology-10-00609-s001.zip › Supplementary biology-1272422/Figure S1. Morphological characteristics of iPSCs.pdf]

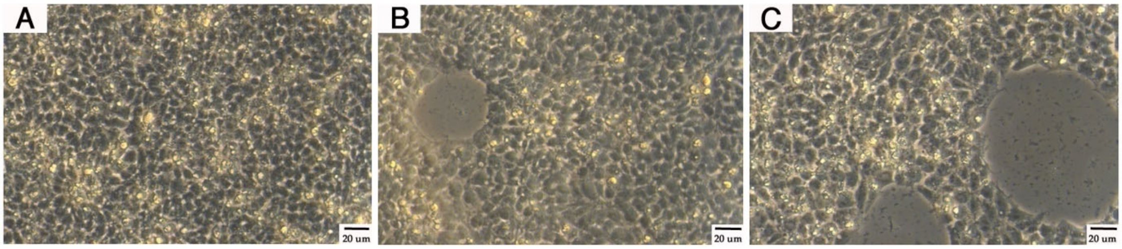

Figure S1. Morphological characteristics of iPSCs. Representative phase contrast microscopy images of the Eu-iPSCs (DS2U clone, **A**), and of the T21-iPSCs (DS1 clone, **B** and DS4 clone, **C**). Scale bar: 20 μm
