# Supplementary material for: Human Trisomic iPSCs from Down Syndrome Fibroblasts Manifest Mitochondrial Alterations Early during Neuronal Differentiation
Source: Biology (Basel). 2021 Jun 30;10(7):609. doi: 10.3390/biology10070609 (PMC8301075; doi:10.3390/biology10070609)
Supplement: Supplementary file 1 [file biology-10-00609-s001.zip › Supplementary biology-1272422/Figure S2. Karyotype of Eu-iPSC and T21-iPSC clones.pdf]

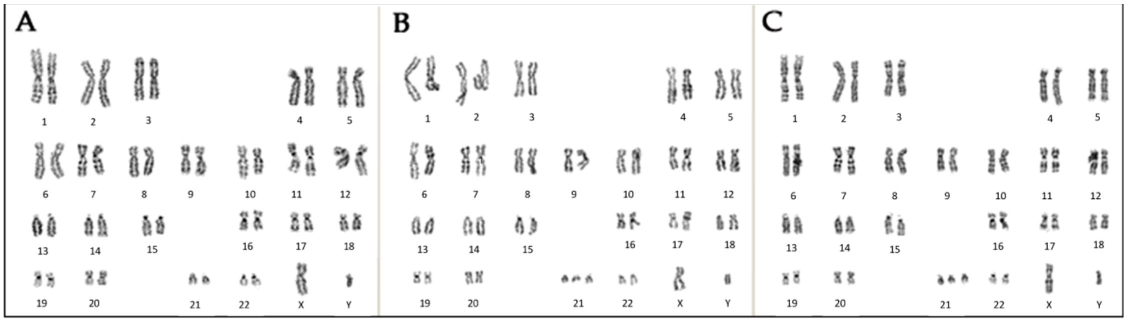

Figure S2. Karyotype of Eu-iPSC and T21-iPSC clones. Karyotype analysis indicates that the Eu-iPSC clone DS2U is disomic (**A**) whereas the T21-iPSC clones DS1 and DS4 are trisomic for chromosome 21 (**B**, **C**). No other chromosomal alteration was detected.
