# Supplementary material for: Human Trisomic iPSCs from Down Syndrome Fibroblasts Manifest Mitochondrial Alterations Early during Neuronal Differentiation
Source: Biology (Basel). 2021 Jun 30;10(7):609. doi: 10.3390/biology10070609 (PMC8301075; doi:10.3390/biology10070609)
Supplement: Supplementary file 1 [file biology-10-00609-s001.zip › Supplementary biology-1272422/Figure S3. Expression of stemness and differentiation markers in iPSC-derived cells after 5 days of neural induction.pdf]

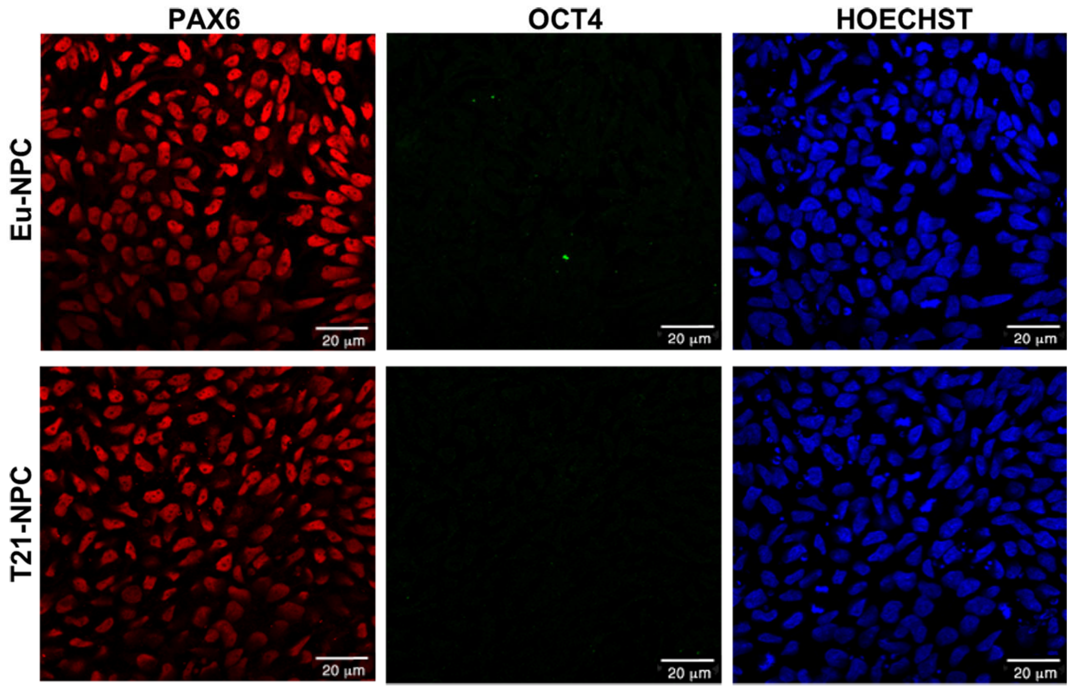

Figure S3. Expression of stemness and differentiation markers in iPSC-derived cells after 5 days of neural induction. Expression of OCT4 and PAX6 was assessed by immunofluorescence in iPSCs after 5 days of culture in differentiation medium. T21-NPC images are representative of the two trisomic clones. Both euploid and trisomic cells are negative for OCT4 (green) and positive for PAX6 (red) staining. Nuclei are also stained with Hoechst (blue). Bar = 20 μm
