# Supplementary material for: Human Trisomic iPSCs from Down Syndrome Fibroblasts Manifest Mitochondrial Alterations Early during Neuronal Differentiation
Source: Biology (Basel). 2021 Jun 30;10(7):609. doi: 10.3390/biology10070609 (PMC8301075; doi:10.3390/biology10070609)
Supplement: Supplementary file 1 [file biology-10-00609-s001.zip › Supplementary biology-1272422/Table S1 Enrichment of genes mapping to chr21 in the list of genes upregulated in T21-iPSCs from EnrichR analysis.pdf]

Table S1. Enrichment of genes mapping to chr21 in the list of genes upregulated in T21-iPSCs from EnrichR analysis

Term	Overlap	P-value	Adjusted P-value	Combined Score	Genes
chr21	81/365	1.50e-17	4.81e-16	132.04	CLIC6;SETD4;COL18A1;RRP1;ITSN1;WDR4;MRPL39;ADARB1;PWP2;BACH1;ETS2;FTCD;CHAF1B;POFUT2;EVA1C;C21ORF91;HMGN1;CBR1;CXADR;IFNGR2;RIPK4;C21ORF58;OLIG1;URB1;MRPS6;LSS;DOPEY2;RIPPLY3;PCP4;CRYZL1;MCM3APAS1;GART;ABCG1;PDE9A;IFNAR1;CSTB;RWDD2B;IGSF5;PDXK;USP16;WRB;PRDM15;ATP5J;TMPRSS2;U2AF1;PTTG1IP;ATP5O;HSPA13;DSCR3;YBEY;HLCS;CBS;CCT8;NDUFV3;CYYR1;PCNT;SLC19A1;SH3BGR;RBM11;CBR3;USP25;C21ORF33;KCNJ6;SLC37A1;IL10RB;TMEM50B;TRAPPC10;MX1;LOC642852;UBE2G2;RRP1B;B3GALT5;SCAF4;SOD1;MCM3AP;RSPH1;MIS18A;SYNJ1;PSMG1;C21ORF2;PAXBP1

For the chromosome positional gene set collection, chromosome 21 genes were significantly enriched among genes upregulated in T21-iPSC public expression data (81 upregulated genes out of the 365 genes).
