# Supplementary material for: Human Trisomic iPSCs from Down Syndrome Fibroblasts Manifest Mitochondrial Alterations Early during Neuronal Differentiation
Source: Biology (Basel). 2021 Jun 30;10(7):609. doi: 10.3390/biology10070609 (PMC8301075; doi:10.3390/biology10070609)
Supplement: Supplementary file 1 [file biology-10-00609-s001.zip › Supplementary biology-1272422/Table S2 GO Biological Process categories of mitochondria related genes dysregulated in iPSCs derived from DS subjects.pdf]

Table S2. GO Biological Process categories of mitochondria related genes dysregulated in iPSCs derived from DS subjects. The most interesting GO terms are highlighted in bold.

Term	Overlap	Genes
**mitochondrion organization (GO:0007005)**	**33/167**	**PIF1;TIMM13;SPG7;MIEF1;MFF;TOMM20;TRAK2;CHCHD2;RHOT1;TIMM17A;MPV17;USP30;DNM1L;FBXO7;PNPT1;NUBPL;BNIP3;SIRT5;TIMM23;TIMM44;TFB2M;ATAD3C;RAB32;AFG3L2;GLUD1;IMMP1L;PINK1;WDR81;YME1L1;TFAM;CYCS;RAB38;SLC25A4**
mitochondrial transport (GO:0006839)	27/135	SLC25A3;TIMM13;ATP5J;SPG7;ATP5O;MFF;TOMM20;ATP5G1;CHCHD4;SLC25A26;GRPEL1;UCP3;ATP5D;TIMM17A;SLC25A20;FBXO7;SLC25A24;PNPT1;BNIP3;TIMM23;TIMM44;SLC25A16;PINK1;BCL2;SLC25A10;SLC25A4;SLC25A14
**cellular response to oxidative stress (GO:0034599)**	**19/115**	**PNPT1;TXNRD1;MPV17L;GSR;HTRA2;PRDX6;DHRS2;TXN2;SOD1;PINK1;PXMP2;CAT;ABL1;CYCS;MPV17;MAPT;SNCA;MAPK3;SLC25A24**
**mitochondrial respiratory chain complex assembly (GO:0033108)**	**14/97**	**COX19;NDUFB9;NDUFA11;NUBPL;NDUFB1;IMMP1L;SCO2;NDUFAF5;NDUFS4;SDHAF1;NDUFAF3;TMEM126B;NDUFV3;NDUFV2**
protein targeting to mitochondrion (GO:0006626)	11/54	CHCHD4;IMMP1L;GRPEL1;TIMM17A;TIMM13;TIMM23;TIMM44;UQCRC2;MFF;TOMM20;FBXO7
mitochondrial transmembrane transport (GO:1990542)	9/32	AFG3L2;MCUR1;CPT1A;SMDT1;UCP3;TIMM13;SLC25A20;MICU1;MCU
respiratory electron transport chain (GO:0022904)	13/94	NDUFB9;NDUFA11;NDUFB1;ETFA;SDHD;COX7A2L;NDUFS4;UQCRC1;CYCS;UQCRC2;NDUFV3;NDUFV2;COX10
establishment of protein localization to mitochondrion (GO:0072655)	10/49	CHCHD4;PINK1;GRPEL1;TIMM17A;TIMM13;TIMM23;TIMM44;MFF;TOMM20;FBXO7
fatty acid beta-oxidation (GO:0006635)	10/50	HADHA;ACAA2;ECI2;ECHDC1;ETFA;ACADM;HADH;ACADS;HIBCH;ACAT1
**mitochondrial ATP synthesis coupled electron transport (GO:0042775)**	**12/85**	**NDUFB9;COX7A2L;NDUFA11;NDUFS4;UQCRC1;NDUFB1;CYCS;SDHD;UQCRC2;NDUFV3;NDUFV2;COX10**
cellular respiration (GO:0045333)	10/57	COX19;NDUFS4;UQCRC1;CYCS;ETFA;UQCRC2;NDUFV2;COQ10B;SLC25A14;COX10
**mitochondrial calcium ion transmembrane transport (GO:0006851)**	**7/21**	**AFG3L2;MCUR1;SMDT1;YME1L1;SPG7;MICU1;MCU**
negative regulation of mitochondrion organization (GO:0010823)	8/33	SLC35F6;PINK1;ACAA2;BNIP3;MPV17L;MAPT;NOL3;CLU
mitochondrial respiratory chain complex I biogenesis (GO:0097031)	10/64	NDUFB9;NDUFA11;NUBPL;NDUFS4;NDUFAF5;NDUFB1;NDUFAF3;TMEM126B;NDUFV3;NDUFV2
negative regulation of apoptotic signaling pathway (GO:2001234)	10/65	SLC35F6;BCL2L10;PINK1;ACAA2;MPV17L;BCL2;RIPK1;NOL3;CLU;MCL1
inner mitochondrial membrane organization (GO:0007007)	8/36	APOO;AFG3L2;ATP5D;TIMM13;ATP5J;IMMT;ATP5O;ATP5G1
**mitochondrial calcium uptake (GO:0036444)**	**5/9**	**AFG3L2;MCUR1;SMDT1;MICU1;MCU**
**cristae formation (GO:0042407)**	**7/27**	**APOO;AFG3L2;ATP5D;ATP5J;IMMT;ATP5O;ATP5G1**
translational termination (GO:0006415)	11/96	MRPS25;MRPS33;MRPL39;MRPS2;MRPL12;MRPL23;ABCE1;MRPL35;MRRF;MRPL44;DAP3
positive regulation of apoptotic signaling pathway (GO:2001235)	10/76	MOAP1;PINK1;BNIP3;BCL2;HTRA2;G0S2;FAS;RIPK1;DNM1L;MFF
**regulation of mitochondrial fission (GO:0090140)**	**6/19**	**PINK1;BNIP3;MIEF1;MAPT;MFF;DNM1L**
**mitochondrial calcium ion homeostasis (GO:0051560)**	**6/19**	**AFG3L2;MCUR1;SMDT1;C19ORF12;MICU1;MCU**
fatty acid catabolic process (GO:0009062)	9/65	HADHA;ACAA2;PCCB;ECHDC1;ACADM;HADH;ACADS;HIBCH;ACAT1
mitochondrial translation (GO:0032543)	11/107	MRPS25;MRPS33;MRPL39;MRPS2;MRPL12;MRPL23;HARS2;MRPL35;MRRF;DAP3;MRPL44
apoptotic mitochondrial changes (GO:0008637)	7/34	BNIP3;CAMK2A;IFI6;BCL2;MFF;DNM1L;CLU
fatty acid oxidation (GO:0019395)	8/50	HADHA;ACAA2;ECHDC1;ACADM;HADH;ACADS;HIBCH;ACAT1
mitochondrial translational termination (GO:0070126)	10/89	MRPS25;MRPS33;MRPL39;MRPS2;MRPL12;MRPL23;MRPL35;MRRF;MRPL44;DAP3

mitochondrion disassembly (GO:0061726)	7/38	FUNDC2;GABARAPL1;PINK1;BNIP3;USP30;FBXO7;ATG13
**positive regulation of mitochondrial fission (GO:0090141)**	**5/14**	**PINK1;BNIP3;MIEF1;MFF;DNM1L**
**autophagy of mitochondrion (GO:0000422)**	**7/40**	**FUNDC2;GABARAPL1;PINK1;BNIP3;USP30;FBXO7;ATG13**
regulation of release of cytochrome c from mitochondria (GO:0090199)	7/40	MOAP1;PINK1;BNIP3;NOL3;MFF;DNM1L;CLU
intrinsic apoptotic signaling pathway (GO:0097193)	10/101	MOAP1;STK11;BCL2L10;BNIP3;ABL1;BCL2;HTRA2;CYCS;PDK1;MCL1
**response to reactive oxygen species (GO:0000302)**	**8/60**	**TXNRD1;MPV17L;CAT;PXMP2;MPV17;MAPT;MAPK3;SOD1**
protein transmembrane import into intracellular organelle (GO:0044743)	7/43	CHCHD4;GRPEL1;TIMM13;TIMM17A;TIMM23;TIMM44;TOMM20
protein oligomerization (GO:0051259)	14/217	OAT;PNPT1;SHMT2;ATPIF1;IDE;NOL3;MFF;NBR1;CAT;YME1L1;RIPK1;MAPT;MICU1;DNM1L
mitochondrial gene expression (GO:0140053)	7/45	PNPT1;MRPS2;TFAM;TFB2M;MRPL12;MRPL23;HARS2
mitochondrial translational elongation (GO:0070125)	9/87	MRPS25;MRPS33;MRPL39;MRPS2;MRPL12;MRPL23;MRPL35;MRPL44;DAP3
cellular response to DNA damage stimulus (GO:0006974)	17/329	MOAP1;FEN1;LIG1;XRCC3;HTRA2;XPC;UNG;STK11;BCL2L10;RAD51C;ALKBH1;ABL1;BCL2;CDK1;UBA1;MCL1;MAPK3
release of cytochrome c from mitochondria (GO:0001836)	5/18	IFI6;BCL2;MFF;DNM1L;CLU
regulation of mitochondrial membrane permeability (GO:0046902)	5/18	BNIP3;CAMK2A;BCL2;SPG7;SLC25A4
cofactor biosynthetic process (GO:0051188)	6/31	NFU1;HSPA9;SLC25A16;NUBPL;IBA57;MMADHC
nucleobase-containing small molecule interconversion (GO:0015949)	6/31	DTYMK;NME6;TXNRD1;GSR;NME4;TYMS
extrinsic apoptotic signaling pathway (GO:0097191)	8/67	MOAP1;BCL2L10;KIAA0141;BCL2;G0S2;FAS;RIPK1;MCL1
regulation of mitochondrion organization (GO:0010821)	7/48	PINK1;RHOT1;ATPIF1;HTRA2;MAPT;MFF;DNM1L
**mitochondrion morphogenesis (GO:0070584)**	**4/9**	**PNPT1;NUBPL;MFF;DNM1L**
mitochondrial protein processing (GO:0034982)	4/9	AFG3L2;IMMP1L;YME1L1;UQCRC2
protein import into mitochondrial matrix (GO:0030150)	5/19	GRPEL1;TIMM17A;TIMM23;TIMM44;TOMM20
regulation of mitochondrial membrane potential (GO:0051881)	6/35	PINK1;ATPIF1;ABL1;BCL2;MAPT;SOD1
regulation of apoptotic process (GO:0042981)	28/815	MTCH2;KIAA0141;HTRA2;NOL3;CLU;BCL2L10;C1QBP;ABL1;HS1BP3;RIPK1;VHL;DNM1L;MCL1;SNCA;HSPA9;MOAP1;BNIP3;NME4;DHRS2;SOD1;PINK1;RPS6KB1;CAT;BCL2;CDK1;YME1L1;FAS;TNFRSF25
**cytosolic calcium ion transport (GO:0060401)**	**5/22**	**AFG3L2;MCUR1;SMDT1;MICU1;MCU**
cell part morphogenesis (GO:0032990)	4/11	PNPT1;NUBPL;MFF;DNM1L
**ATP metabolic process (GO:0046034)**	**7/56**	**ATP5D;UQCRC1;ATP5J;UQCRC2;ATP5O;ATP5G1;CLPX**
cellular amino acid catabolic process (GO:0009063)	5/23	ACAD8;BCKDHB;ABAT;HIBCH;ACAT1
translational elongation (GO:0006414)	9/105	MRPS25;MRPS33;MRPL39;MRPS2;MRPL12;MRPL23;MRPL35;DAP3;MRPL44
negative regulation of intrinsic apoptotic signaling pathway (GO:2001243)	7/62	BCL2L10;PINK1;BCL2;HTRA2;NOL3;CLU;MCL1
tetrahydrofolate metabolic process (GO:0046653)	4/13	DHFR;SHMT2;ATPIF1;TYMS
positive regulation of release of cytochrome c from mitochondria (GO:0090200)	5/26	MOAP1;PINK1;BNIP3;MFF;DNM1L
metallo-sulfur cluster assembly (GO:0031163)	4/14	HSPA9;NFU1;NUBPL;IBA57
calcium ion import (GO:0070509)	5/28	AFG3L2;MCUR1;SMDT1;MICU1;MCU
tRNA aminoacylation for protein translation (GO:0006418)	6/46	PARS2;MARS2;MRPL39;DARS2;GARS;HARS2

**mitochondrial electron transport. NADH to ubiquinone (GO:0006120)**	**6/46**	**NDUFB9;NDUFA11;NDUFS4;NDUFB1;NDUFV3;NDUFV2**
regulation of mitochondrial translation (GO:0070129)	4/15	ALKBH1;C1QBP;NSUN3;MALSU1
coenzyme biosynthetic process (GO:0009108)	5/29	DHFR;SLC25A16;ATPIF1;MMADHC;ACAT1
intrinsic apoptotic signaling pathway in response to DNA damage (GO:0008630)	6/48	MOAP1;BCL2L10;ABL1;BCL2;HTRA2;MCL1
positive regulation of mitochondrion organization (GO:0010822)	9/128	MOAP1;PINK1;BNIP3;HTRA2;MIEF1;DNM1L;MFF;FBXO7;ATG13
regulation of lipid metabolic process (GO:0019216)	8/100	LACTB;SLC27A1;CPT1A;ACSL1;TXNRD1;ME1;G0S2;ACADM
negative regulation of mitochondrial outer membrane permeabilization involved in apoptotic signaling pathway (GO:1901029)	3/7	SLC35F6;ACAA2;MPV17L
adenine nucleotide transport (GO:0051503)	3/7	SLC25A16;SLC25A4;SLC25A24
purine ribonucleotide transport (GO:0015868)	3/7	SLC25A16;SLC25A4;SLC25A24
**microglial cell activation (GO:0001774)**	**3/7**	**MAPT;CLU;SNCA**
iron-sulfur cluster assembly (GO:0016226)	4/18	HSPA9;NFU1;NUBPL;IBA57
regulation of cellular response to stress (GO:0080135)	8/104	CHCHD2;PINK1;PNPT1;CAMK2A;ABL1;MAPT;MCL1;MAPK3
activation of cysteine-type endopeptidase activity involved in apoptotic process (GO:0006919)	7/79	BCL2L10;IFI27;FAS;CYCS;RIPK1;MAPT;SNCA
extrinsic apoptotic signaling pathway in absence of ligand (GO:0097192)	4/19	MOAP1;BCL2L10;BCL2;MCL1
mitochondrial membrane organization (GO:0007006)	4/19	BNIP3;BCL2;SPG7;SLC25A4
DNA metabolic process (GO:0006259)	14/314	PIF1;FEN1;ENDOG;LIG1;XRCC3;XPC;TYMS;UNG;POLD4;RAD51C;ALKB H1;CDK1;DNA2;POLG
positive regulation of cell death (GO:0010942)	6/57	BNIP3;HTRA2;RIPK1;MAPT;CLU;SNCA
positive regulation of cysteine-type endopeptidase activity involved in apoptotic process (GO:0043280)	8/109	BCL2L10;IFI27;HTRA2;CYCS;FAS;RIPK1;MAPT;SNCA
regulation of stress-activated protein kinase signaling cascade (GO:0070302)	3/8	NBR1;FAS;MAPK3
regulation of establishment of protein localization to mitochondrion (GO:1903747)	3/8	PINK1;ATPIF1;MAPT
positive regulation of mitochondrial calcium ion concentration (GO:0051561)	3/8	MCUR1;MICU1;MCU
short-chain fatty acid catabolic process (GO:0019626)	3/8	PCCB;ACADS;PCK2
hydrogen peroxide metabolic process (GO:0042743)	4/20	MAOB;CAT;PRDX6;SOD1
mitochondrial RNA metabolic process (GO:0000959)	4/20	PNPT1;TFAM;TFB2M;MRPL12
negative regulation of apoptotic process (GO:0043066)	18/485	HSPA9;BNIP3;AGAP2;IFI6;NOL3;DHRS2;SOD1;BCL2L10;PINK1;RPS6KB1; CAT;BCL2;CDK1;YME1L1;FAS;VHL;MCL1;SNCA
**negative regulation of neuron death (GO:1901215)**	**7/83**	**PINK1;AGAP2;BCL2;HTRA2;FBXO7;SNCA;SOD1**
branched-chain amino acid catabolic process (GO:0009083)	4/21	ACAD8;BCKDHB;HIBCH;ACAT1
mitochondrial ATP synthesis coupled proton transport (GO:0042776)	4/21	ATP5D;ATP5J;ATP5O;ATP5G1
**calcium ion transmembrane transport (GO:0070588)**	**7/86**	**AFG3L2;MCUR1;SMDT1;YME1L1;SPG7;MICU1;MCU**
DNA biosynthetic process (GO:0071897)	5/39	POLD4;RAD51C;XRCC3;DNA2;TYMS
mitochondrial fragmentation involved in apoptotic process (GO:0043653)	3/9	BNIP3;MFF;DNM1L
cellular protein catabolic process (GO:0044257)	6/62	BNIP3;YME1L1;HTRA2;RIPK1;IDE;CLPX

branched-chain amino acid metabolic process (GO:0009081)	4/22	ACAD8;BCKDHB;HIBCH;ACAT1
negative regulation of growth (GO:0045926)	8/120	TCHP;CYP27B1;STK11;PNPT1;NME6;PPP2R1A;SLIT3;ESR2
cellular response to reactive oxygen species (GO:0034614)	6/65	MPV17L;PXMP2;ABL1;MPV17;MAPT;MAPK3
glycine metabolic process (GO:0006544)	3/10	GCSH;SHMT2;GCAT
negative regulation of oxidative stress-induced cell death (GO:1903202)	4/24	PINK1;HTRA2;NOL3;FBXO7
respiratory chain complex IV assembly (GO:0008535)	4/25	COX19;SCO2;COX11;COX10
long-chain fatty acid transport (GO:0015909)	4/25	SLC27A1;CPT1A;ACSL1;SLC25A20
carnitine metabolic process (GO:0009437)	3/11	CPT1A;TMLHE;ACADM
mitochondrion distribution (GO:0048311)	3/11	MAPT;DNM1L;TRAK2
oxidative phosphorylation (GO:0006119)	3/11	ATP5D;UQCRC1;UQCRC2
regulation of intrinsic apoptotic signaling pathway (GO:2001242)	5/47	BCL2L10;PINK1;BCL2;DNM1L;MCL1
ATP synthesis coupled proton transport (GO:0015986)	4/27	ATP5D;ATP5J;ATP5O;ATP5G1
regulation of extrinsic apoptotic signaling pathway in absence of ligand (GO:2001239)	4/27	IFI6;BCL2;HTRA2;MCL1
purine ribonucleoside triphosphate biosynthetic process (GO:0009206)	4/27	ATP5D;ATP5J;ATP5O;ATP5G1
**regulation of autophagy of mitochondrion (GO:1903146)**	**4/27**	**PINK1;HTRA2;DNM1L;FBXO7**
mitochondrial outer membrane permeabilization (GO:0097345)	3/12	MOAP1;RHOT1;BNIP3
transcription from mitochondrial promoter (GO:0006390)	3/12	TFAM;TFB2M;MRPL12
protein targeting (GO:0006605)	8/133	TIMM17A;TIMM13;TIMM23;TIMM44;MFF;TOMM20;FBXO7;TRAK2
**regulation of autophagy (GO:0010506)**	**10/203**	**STK11;PINK1;BNIP3;ABL1;BCL2;HTRA2;MAPT;DNM1L;ATG13;MCL1**
aerobic respiration (GO:0009060)	4/28	COX19;UQCRC1;UQCRC2;SLC25A14
regulation of reactive oxygen species metabolic process (GO:2000377)	5/49	PINK1;SIRT5;RIPK1;MPV17;SNCA
negative regulation of programmed cell death (GO:0043069)	15/408	HSPA9;BNIP3;NOL3;DHRS2;BCL2L10;RPS6KB1;CAT;BCL2;CDK1;YME1L1;FAS;VHL;SLC25A4;MCL1;SNCA
negative regulation of extrinsic apoptotic signaling pathway (GO:2001237)	6/76	IFI6;BCL2;FAS;RIPK1;NOL3;MCL1
response to iron ion (GO:0010039)	3/13	SLC11A2;BCL2;SNCA
mitochondrial genome maintenance (GO:0000002)	3/13	PIF1;MPV17;SLC25A4
water-soluble vitamin metabolic process (GO:0006767)	6/77	DHFR;CYB5R3;PC;SHMT2;PCCB;MMADHC
DNA repair (GO:0006281)	12/288	POLD4;FEN1;RAD51C;LIG1;ALKBH1;XRCC3;ABL1;CDK1;XPC;DNA2;PARP9;UNG
regulation of primary metabolic process (GO:0080090)	8/139	LACTB;SLC27A1;CPT1A;ACSL1;TXNRD1;ME1;G0S2;ACADM
ubiquinone metabolic process (GO:0006743)	3/14	COQ10B;COQ6;COQ5
mitochondrial electron transport. ubiquinol to cytochrome c (GO:0006122)	3/14	UQCRC1;CYCS;UQCRC2
quinone biosynthetic process (GO:1901663)	3/14	COQ10B;COQ6;COQ5
necroptotic process (GO:0070266)	3/14	PGAM5;RIPK1;DNM1L
membrane fusion (GO:0061025)	5/54	AFG3L2;USP30;DNM1L;MFF;NAPG
positive regulation of programmed cell death (GO:0043068)	11/257	MOAP1;MTCH2;C1QBP;BNIP3;ABL1;FAS;HTRA2;RIPK1;DNM1L;SNCA;SOD1
ATP biosynthetic process (GO:0006754)	4/32	ATP5D;ATP5J;ATP5O;ATP5G1

mismatch repair (GO:0006298)	4/32	POLD4;LIG1;ABL1;XPC
regulation of organelle organization (GO:0033043)	7/113	CLIC4;PINK1;RHOT1;MAPT;DNM1L;MFF;MAPK3
positive regulation of developmental process (GO:0051094)	7/113	PINK1;C1QBP;BNIP3;MIEF1;VHL;DNM1L;MFF
ubiquinone biosynthetic process (GO:0006744)	3/15	COQ10B;COQ6;COQ5
fatty acid beta-oxidation using acyl-CoA dehydrogenase (GO:0033539)	3/15	ETFA;ACADM;ACADS
positive regulation of cysteine-type endopeptidase activity involved in apoptotic signaling pathway (GO:2001269)	3/15	HTRA2;FAS;RIPK1
negative regulation of cell growth (GO:0030308)	7/115	TCHP;CYP27B1;STK11;NME6;PPP2R1A;SLIT3;ESR2
positive regulation of apoptotic process (GO:0043065)	12/307	MOAP1;MTCH2;C1QBP;BNIP3;ABL1;FAS;HTRA2;CYCS;RIPK1;DNM1L;SNCA;SOD1
**mitochondrial fusion (GO:0008053)**	**3/16**	**AFG3L2;USP30;MFF**
programmed necrotic cell death (GO:0097300)	3/16	PGAM5;RIPK1;DNM1L
protein homooligomerization (GO:0051260)	9/190	PNPT1;ATPIF1;CAT;HTRA2;RIPK1;IDE;DNM1L;MICU1;MFF
apoptotic process (GO:0006915)	10/231	TCHP;STK11;PPP2R1A;BNIP3;BCL2;FAS;C19ORF12;RIPK1;TNFRSF25;DNM1L
translation (GO:0006412)	10/232	MRPS33;MARS2;MRPS2;DARS2;GARS;MRPL23;ABCE1;HARS2;MRPL35;MRRF
regulation of mitochondrial outer membrane permeabilization involved in apoptotic signaling pathway (GO:1901028)	3/17	SLC35F6;ACAA2;MPV17L
negative regulation of intracellular transport (GO:0032387)	3/17	SLC35F6;ACAA2;MPV17L
macrophage activation (GO:0042116)	3/17	MAPT;CLU;SNCA
base-excision repair (GO:0006284)	4/37	LIG1;DNA2;UNG;POLG
organelle fusion (GO:0048284)	3/18	AFG3L2;USP30;MFF
regulation of oxidative stress-induced intrinsic apoptotic signaling pathway (GO:1902175)	3/18	HTRA2;NOL3;SOD1
negative regulation of oxidative stress-induced intrinsic apoptotic signaling pathway (GO:1902176)	3/18	PINK1;HTRA2;NOL3
**regulation of cell growth (GO:0001558)**	**9/201**	**TCHP;CYP27B1;STK11;NME6;NCBP1;PPP2R1A;BCL2;SLIT3;ESR2**
purine ribonucleoside monophosphate biosynthetic process (GO:0009168)	4/39	ATP5D;ATP5J;ATP5O;ATP5G1
tRNA aminoacylation (GO:0043039)	4/40	MARS2;DARS2;GARS;HARS2
negative regulation of membrane potential (GO:0045837)	2/6	BNIP3;MAPT
regulation of protein targeting (GO:1903533)	2/6	PINK1;ATPIF1
regulation of cellular response to oxidative stress (GO:1900407)	2/6	DHFR;PINK1
pyrimidine deoxyribonucleotide biosynthetic process (GO:0009221)	2/6	DTYMK;TYMS
folic acid-containing compound biosynthetic process (GO:0009396)	2/6	DHFR;ATPIF1
positive regulation of oxidative stress-induced cell death (GO:1903209)	2/6	RIPK1;SOD1
peptidyl-serine autophosphorylation (GO:0036289)	2/6	PINK1;RIPK1
glutamate catabolic process (GO:0006538)	2/6	GLUD1;GLUL
protein processing (GO:0016485)	6/101	AFG3L2;IMMP1L;UQCRC1;YME1L1;HTRA2;UQCRC2
phosphorylation (GO:0016310)	13/386	CAMK2A;GK5;RPS6KA6;STK11;PINK1;RPS6KB1;ATP5D;UQCRC1;ABL1;CDK1;PICK1;UQCRC2;MAPK3

mitochondrial electron transport. cytochrome c to oxygen (GO:0006123)	3/21	COX7A2L;CYCS;COX10
**mitochondrial fission (GO:0000266)**	**3/21**	**MIEF1;MFF;DNM1L**
glutamate metabolic process (GO:0006536)	3/21	GLUD1;OAT;GLUL
carboxylic acid biosynthetic process (GO:0046394)	4/44	GLUD1;OAT;ATPIF1;GLUL
response to metal ion (GO:0010038)	5/72	SLC11A2;ATP5D;BCL2;MAPT;SNCA
positive regulation of proteolysis involved in cellular protein catabolic process (GO:1903052)	3/22	SMURF1;ATPIF1;CLU
negative regulation of autophagy (GO:0010507)	4/46	PINK1;BCL2;MCL1;SNCA
cellular response to catecholamine stimulus (GO:0071870)	3/23	ABL1;MAPK3;SNCA
regulation of proteolysis involved in cellular protein catabolic process (GO:1903050)	3/23	PINK1;ATPIF1;UCHL5
negative regulation of signal transduction in absence of ligand (GO:1901099)	3/23	IFI6;BCL2;MCL1
folic acid-containing compound metabolic process (GO:0006760)	3/23	DHFR;SHMT2;ATPIF1
negative regulation of extrinsic apoptotic signaling pathway in absence of ligand (GO:2001240)	3/23	IFI6;BCL2;MCL1
rRNA methylation (GO:0031167)	3/23	NSUN3;TFB2M;MRM1
negative regulation of cellular amide metabolic process (GO:0034249)	5/75	DHFR;MALSU1;TYMS;CLU;SNCA
purine ribonucleotide biosynthetic process (GO:0009152)	5/75	ATP5D;ATP5J;ATP5O;ATP5G1;ACAT1
intracellular sterol transport (GO:0032366)	2/7	SERAC1;ABCG1
mitochondrial DNA metabolic process (GO:0032042)	2/7	DNA2;POLG
regulation of mitochondrial membrane permeability involved in apoptotic process (GO:1902108)	2/7	ACAA2;CAMK2A
pteridine-containing compound biosynthetic process (GO:0042559)	2/7	DHFR;ATPIF1
**positive regulation of autophagy of mitochondrion (GO:1903599)**	**2/7**	**ATPIF1;FBXO7**
strand invasion (GO:0042148)	2/7	RAD51C;XRCC3
cellular lipid biosynthetic process (GO:0097384)	2/7	AGPS;ACAT1
negative regulation of transporter activity (GO:0032410)	2/7	NDFIP2;SNCA
tetrahydrofolate interconversion (GO:0035999)	2/7	ATPIF1;TYMS
G-quadruplex DNA unwinding (GO:0044806)	2/7	PIF1;DNA2
regulation of programmed cell death (GO:0043067)	10/268	MOAP1;PINK1;BNIP3;ABL1;FAS;NME4;HS1BP3;RIPK1;TNFRSF25;CLU
**macroautophagy (GO:0016236)**	**6/110**	**GABARAPL1;PINK1;NBR1;PGAM5;TOMM20;ATG13**
RNA methylation (GO:0001510)	4/49	NSUN3;TFB2M;MRM1;GTPBP3
DNA recombinase assembly (GO:0000730)	2/8	RAD51C;XRCC3
myelin maintenance (GO:0043217)	2/8	CLU;SOD1
amyloid fibril formation (GO:1990000)	2/8	RIPK1;MAPT
lagging strand elongation (GO:0006273)	2/8	LIG1;DNA2
DNA repair complex assembly (GO:0090735)	2/8	RAD51C;XRCC3
negative regulation of monooxygenase activity (GO:0032769)	2/8	CYP27B1;SNCA

DNA damage induced protein phosphorylation (GO:0006975)	2/8	ABL1;MAPK3
negative regulation of establishment of protein localization (GO:1904950)	2/8	NDFIP2;MAPT
double-strand break repair via synthesis-dependent strand annealing (GO:0045003)	2/8	RAD51C;XRCC3
positive regulation of mitochondrial membrane permeability involved in apoptotic process (GO:1902110)	2/8	RHOT1;BNIP3
tRNA aminoacylation for mitochondrial protein translation (GO:0070127)	2/8	DARS2;GARS
negative regulation of cell death (GO:0060548)	5/81	PINK1;BNIP3;HTRA2;CLU;SNCA
strand displacement (GO:0000732)	3/26	RAD51C;XRCC3;DNA2
protein maturation (GO:0051604)	5/83	GCSH;NFU1;AFG3L2;UQCRC1;UQCRC2
