# Supplementary material for: Human Trisomic iPSCs from Down Syndrome Fibroblasts Manifest Mitochondrial Alterations Early during Neuronal Differentiation
Source: Biology (Basel). 2021 Jun 30;10(7):609. doi: 10.3390/biology10070609 (PMC8301075; doi:10.3390/biology10070609)
Supplement: Supplementary file 1 [file biology-10-00609-s001.zip › Supplementary biology-1272422/Table S4 mTORC1 signaling enriched of genes upregulated in T21-iPSC from EnrichR analysis.pdf]

Table S4. mTORC1 signaling enriched of genes upregulated in T21-iPSC from EnrichR analysis.

Term	Overlap	P Value	Adjusted P Value	Combined Score	Genes
mTORC1 Signaling	37/200	0.0000012	0.00006	36.17	SHMT2;USO1;STC1;CXCR4;SLC7A11;SLC1A5;ADD3;CORO1A;HK2;HSP90B1;GGA2;SLC9A3R1;STIP1;NFIL3;ME1;PHGDH;PSPH;HSPA9;EGLN3;HSPA5;HSPA4;IFRD1;ASNS;DHCR24;TUBA4A;SLC7A5;GCLC;UFM1;PSAT1;PSME3;CTH;CCNG1;CD9;PSMG1;SQSTM1;PPIA;SKAP2

The mTORC1 signaling was the pathway most enriched of genes upregulated in T21-iPSC public expression data (37 dysregulated genes out of the 200 genes involved in the pathway)
