# Supplementary material for: Human Trisomic iPSCs from Down Syndrome Fibroblasts Manifest Mitochondrial Alterations Early during Neuronal Differentiation
Source: Biology (Basel). 2021 Jun 30;10(7):609. doi: 10.3390/biology10070609 (PMC8301075; doi:10.3390/biology10070609)
Supplement: Supplementary file 1 [file biology-10-00609-s001.zip › Supplementary biology-1272422/TABLES~3.pdf]

Table S3: GO cellular component category for 'Integral component of mitochondrial membrane' of DEGs downregulated in T21-iPSCs.

ID: GO:0032592; Name: integral component of mitochondrial membrane		
size=69; overlap=15; expect=4.09; **enrichment Ratio=3.67**; PValue=9.688e-6; FDR=1.899e-4		
**Gene Symbol**	**Gene Name**	**Entrez Gene**
CHCHD6	coiled-coil-helix-coiled-coil-helix domain containing 6	84303
APOOL	apolipoprotein O like	139322
ABCB10	ATP binding cassette subfamily B member 10	23456
SPG7	SPG7, paraplegin matrix AAA peptidase subunit	6687
BNIP3	BCL2 interacting protein 3	664
IMMT	inner membrane mitochondrial protein	10989
MCUR1	mitochondrial calcium uniporter regulator 1	63933
RHOT1	ras homolog family member T1	55288
TOMM20	translocase of outer mitochondrial membrane 20	9804
SMDT1	single-pass membrane protein with aspartate rich tail 1	91689
COX11	cytochrome c oxidase copper chaperone COX11	1353
MFF	mitochondrial fission factor	56947
SLC25A3	solute carrier family 25 member 3	5250
ARMCX3	armadillo repeat containing X-linked 3	51566
MICU1	mitochondrial calcium uptake 1	10367

GO cellular component category for 'Integral component of mitochondrial membrane' was more than 3.6 times enriched of DEGs downregulated in DS iPSCs
